# SATIN: a micro and mini satellite mining tool of total genome and coding regions with analysis of perfect repeats polymorphism in coding regions

**DOI:** 10.1186/s12859-024-05842-2

**Published:** 2024-06-18

**Authors:** Carlos Willian Dias Dantas, Sebastião Rodrigues da Costa Neto, Sandy Ingrid Aguiar Alves, Kenny da Costa Pinheiro, Edian Franklin Franco De Los Santos, Rommel Thiago Jucá Ramos

**Affiliations:** 1 Institute of Biological Sciences, Federal University of Minas Gerais, Belo Horizonte, Brazil; 2 Simulation and Computational Biology Laboratory, High Performance Computing Center, Federal University of Pará, Belém, Brazil; 3 Universidad Tecnológica de Santiago, Campus Central de Herrera, Isabel Aguiar No. 61 21243, Santo Domingo, Dominican Republic

**Keywords:** Microsatellite, Simple sequence repeats, SATIN

## Abstract

**Background:**

Tandem repeats are specific sequences in genomic DNA repeated in tandem that are present in all organisms. Among the subcategories of TRs we have Satellite repeats, that is divided into macrosatellites, minisatellites, and microsatellites, being the last two of specific interest because they can identify polymorphisms between organisms due to their instability. Currently, most mining tools focus on Simple Sequence Repeats (SSR) mining, and only a few can identify SSRs in the coding regions.

**Results:**

We developed a microsatellite mining software called SATIN (Micro and Mini SATellite IdentificatioN tool) based on a new sliding window algorithm written in C and Python. It represents a new approach to SSR mining by addressing the limitations of existing tools, particularly in coding region SSR mining. SATIN is available at https://github.com/labgm/SATIN.git. It was shown to be the second fastest for perfect and compound SSR mining. It can identify SSRs from coding regions plus SSRs with motif sizes bigger than 6. Besides the SSR mining, SATIN can also analyze SSRs polymorphism on coding-regions from pre-determined groups, and identify SSRs differentially abundant among them on a per-gene basis. To validate, we analyzed SSRs from two groups of *Escherichia coli* (K12 and O157) and compared the results with 5 known SSRs from coding regions. SATIN identified all 5 SSRs from 237 genes with at least one SSR on it.

**Conclusions:**

The SATIN is a novel microsatellite search software that utilizes an innovative sliding window technique based on a numerical list for repeat region search to identify perfect, and composite SSRs while generating comprehensible and analyzable outputs. It is a tool capable of using files in fasta or GenBank format as input for microsatellite mining, also being able to identify SSRs present in coding regions for GenBank files. In conclusion, we expect SATIN to help identify potential SSRs to be used as genetic markers.

**Supplementary Information:**

The online version contains supplementary material available at 10.1186/s12859-024-05842-2.

## Background

Tandem repeats or TRs are specific regions of blocks of sequences (motifs) of different sizes in the genetic material that are repeated in tandem in different organisms [1]. These repetitions are non-random and appear important in several organisms [1–3].

TRs are divided into ribosomal DNA and Satellite repeats (SRs). The latter is further classified into 1—Macrosatellites, which are repetitive sequences with the size of several kb in length; 2—Minisatellites, which are repetitive sequences with sizes greater than 10 up to a few hundred nucleotides; and 3— Microsatellites or Simple Sequence Repeats (SSRs), which are smaller repetitive sequences whose repeating unit (motif), ranges from 1 to 6 base pairs [3, 4].

Among Satellite repeats, Mini and microsatellites are highlighted due to their instability and dynamics on populations, being microsatellites way more abundant than the other repeats [4].

Microsatellites or simple sequence repeats (SSRs), are widely used to identify specific molecular sequences in an unknown DNA pool. Microsatellites can be classified, according to the observed repetition pattern, into (I) Perfect microsatellites, which exhibit perfect repetitions of a single motif, e.g., (GC)15; (II) Imperfect microsatellites, which have different bases between the repetition pattern that are not in the motif, e.g., (GT)4A(GT)8 and (III) Compound microsatellites, which contain different motifs (two or more) repeated in tandem, e.g., (TA)8(GC)7 [3, 5].

These sequences are highly suitable for revealing polymorphisms among individuals, which is why they are widely used in population studies, genetic similarity analysis, and distance analysis. Furthermore, microsatellites can be found in eukaryotes, prokaryotes, and viruses, exhibiting a wide distribution through the genome and being present in both genic and intergenic regions. These repeated sequences have mutation rates ranging from 10^3^ to 10⁶ per cell generation, and due to this instability, they are highly relevant in evolutionary studies [3]. Besides, substantial results show that SSRs are nonrandomly distributed in protein-coding regions, and microsatellites within genes are probably subjected to stronger selective pressure than other regions due to their significant functional importance [6]. Since microsatellites occur throughout the genome of different species, they have become suitable for studying genetic diversity among species and populations [7]. Minisatellites can also be highly polymorphic in terms of copy number, length, and composition and can be subjected to variations during meiosis, differently from microsatellites, making them also useful for DNA fingerprinting and population studies [4, 8].

Both microsatellites and minisatellites might have a role in genetic tunning on some genes of eukaryotes and prokaryotes, by the repeat copy number, suggesting that they might act as “tuning knobs” on gene expression based on the number of tandem repeats present [6]. Aside from that, SSRs present in genes have a higher mutation rate than the SSRs in non-genic regions and, for example, in primates, a high rate of polymorphism with elongation/shortening process is an important factor of molecular evolution [6, 9].

Due to their importance, SSRs, algorithms, and search tools have been developed for mining repeat regions to identify and monitor microsatellites as genetic markers in various organisms [10]. Among the different approaches used for microsatellite identification algorithms, these search algorithms can be divided into stochastic and deterministic models, where a stochastic algorithm uses a range of values for each variable, allowing for some randomness. In contrast, a deterministic algorithm uses a single estimate to represent values of all variables, being more predictable [10, 11].

The methods used to identify SSRs and the types of outputs vary among different tools. For example, MISA [12] searches for perfect, and compound repeats through exact searches in keyword trees. IMEx [13] is designed to identify perfect, imperfect, and compound repeats and uses a sliding window search approach to identify indels and substitutions. Alignment-based methods using dynamic programming matrices are employed in STAR [14], Mreps [15], and STRING [16] to define approximate repeats, which can be efficient in finding exact and approximate patterns but may be computationally inefficient and slower due to more intensive calculations.

On the other hand, REPuter [17] and MISA [12, 18] uses Suffix Trees and Keyword Trees, respectively, which are data structures that can locate subsequences in O(p) time complexity, where p is the size of the pattern. However, Suffix Trees can require O(n^2^/2) memory space, where n is the size of the sequence. IMEx [13] performs a two-stage sliding window search. Initially, it searches for sequences that repeat at least twice (e.g., ATC ATC) without imperfections (i.e., k = 0). It extends the search in the edges of the sequence, allowing up to $k$ imperfections [12, 13].

Currently, several researchers need advanced computational training and focus a great amount of time on installing and running the tools for microsatellite mining, without mentioning that some tools have some limitations related to the machine capability and the size of input data intended to be used [19]. Besides, some tools are not memory efficient and therefore, are not capable of analyzing large files, beyond that most SSR mining tools do not generate outputs capable of differentiating SSR present in coding and non-coding regions.

So, we present a new tool for mining microsatellites and minisatellites based on a deterministic sliding window algorithm called SATIN (Micro and Mini SATellite IdentificatioN tool) with an innovative method for Satellite repeats mining that offers lower memory and processing requirements when compared to methods such as suffix trees or dynamic programming.

It offers functionalities for identifying perfect, and compound SSRs, while generating comprehensible and analyzable outputs. It is a tool capable of using files in fasta or GenBank format as input for microsatellite mining, and it is also able to identify SSRs present in coding regions for GenBank files. SATIN is a memory-efficient mini and microsatellite mining tool that can identify SSRs present in coding and non-coding regions without the limitation of motif size.

## Implementation

### Method of the SATIN search algorithm

SATIN’s algorithm is based on the user setup of the motif’s size to be determined as input, with *m* for the motif size (e.g., 3 for ATG ATG), and exponent as the total number of motif repetitions (e.g., 2 for ATG ATG), where *MinExponent* represents the minimum number of repetitions required to be considered as an SSR.

**Fig. 1:**
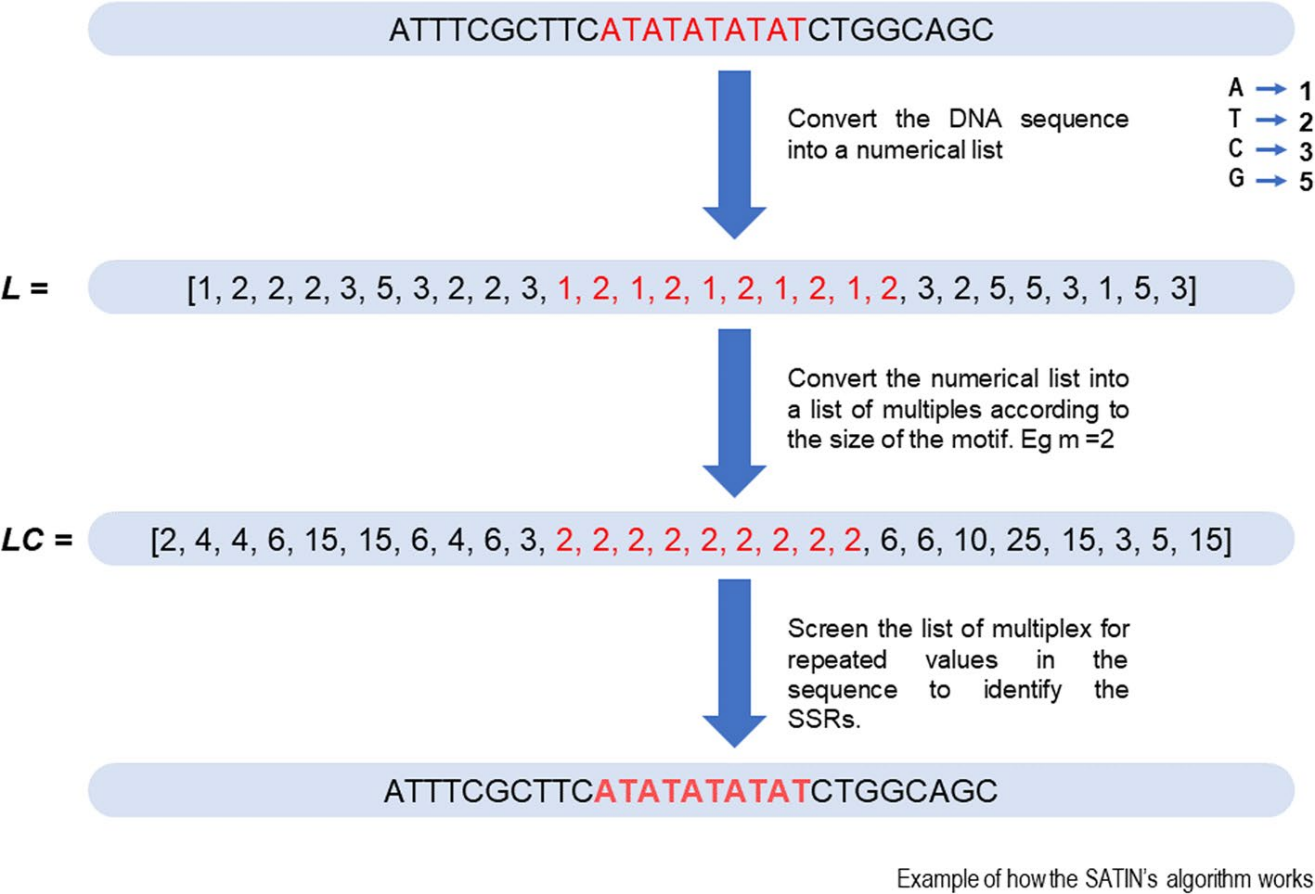
Example of SSRs searching algorithm used in SATIN. A sequence with AT repetitions where the sequence is converted into a numerical list (L) and then into a multiples list (LC) with a motif size of 2 (m). From there, a search mechanism is used on the LC list, considering two neighboring k-mers at a time that repeat in tandem, to identify the SSRs

Initially, the nucleotide sequence is converted into a numeric list $L = L_{i} ... L_{n}$, where each nucleotide corresponds to a prime integer value or the number 1. Subsequently, $L$, a list of composite numbers $LC = LC_{i} ... LC_{n - m + 1}$ is generated, where each value in the set is obtained by multiplying the values of k-mers of size m belonging to $L$. Next, the consecutive repetition of values $LC$ is counted, and whenever the repetition reaches the value of *MinRepMultiples*, a region with a perfect repetition is identified, which may or may not be further extended (Fig. 1).

$$MinRepMultiples = \left( {m*MinExponent} \right) - \left( {m - 1} \right)$$

In practice, the L values of two neighboring k-mers at a time are needed for comparison, which can be calculated by going through m characters for each character in the sequence. As a result, the processing and LC are not created and stored in memory because only the time increases as *m* grows. On the other hand, by converting the k-mer sequences into corresponding integer values, fewer bits are used (for sufficiently large *m*) in comparison to character strings.

When a repetition region is found in LC, satisfying the minimum repeat count, the start and end positions of that region are determined. Finally, the identified perfect SSRs are stored in a matrix (Table 1).

**Table 1:** Matrix of the recorded positions of repeated sequences during the SSRs mining. From left to right, it is the SSR identification order, the size of the identified motif, the number of times this motif repeats (exponent), and the start and end position of the repetition found

SSR id	motif	Exponent	Start position	End position
1	3	2	4	8
2	2	3	9	14
3	2	6	24	35

In the example of searching for microsatellites. When searching for different motif sizes, the matrix will be populated in such a way that it becomes unsorted since each motif type (e.g., mono, di, tri, etc.) is searched one at a time in the sequence. Therefore, a sorting step is necessary to align each repetition found in the matrix based on its start position.

The subsequent steps after sorting involve adding markers to the matrix, which aid in defining the SSRs. One of the functions of the markers is to identify repetitions occurring at the same position. In such cases, the SSRs with the smaller motif are considered (e.g., considering (AC)4 instead of (ACAC)2). As a result, q is the number of perfect SSRs found, and the redundancy removal step is performed in O(q^2^) (Order of q^2^) time.

Finally, the search for compound SSRs is carried out in O(q) time by comparing the positions of adjacent SSRs in the matrix to check if they fall within the accepted distance. For this, the value of the end position of SSR 1 is subtracted from the start position of SSR 2. If the result is a distance less than or equal to 10 for example (int = 10), the positions of both SSRs are updated to negative values, indicating that they belong to a unique tract of compound SSR. Additionally, the integer value of the position is retained to keep the information about the distance between each motif. The same process is applied to SSRs 2 and 3. At the end of this process, a single composite or compound SSR is obtained at the region starting at position 4 and ending at position 35, where before would be considered as 3 distinct SSRs (Table 2).

**Table 2:** Post-processing result matrix of the SSR mining recorded for compound SSRs considering the intervals (int = 10) between identified Satellite repeats

SSR id	motif	Exponent	Start position	End position
1	3	2	4	− 8
2	2	3	− 9	− 14
3	2	6	− 24	35

## Tool overview

SATIN can be used on Linux, and, as input files, SATIN is capable of processing fasta and GenBank file formats. When GenBank files are used, the software will search for repetitive regions throughout the genome and within coding regions.

**Fig. 2:**
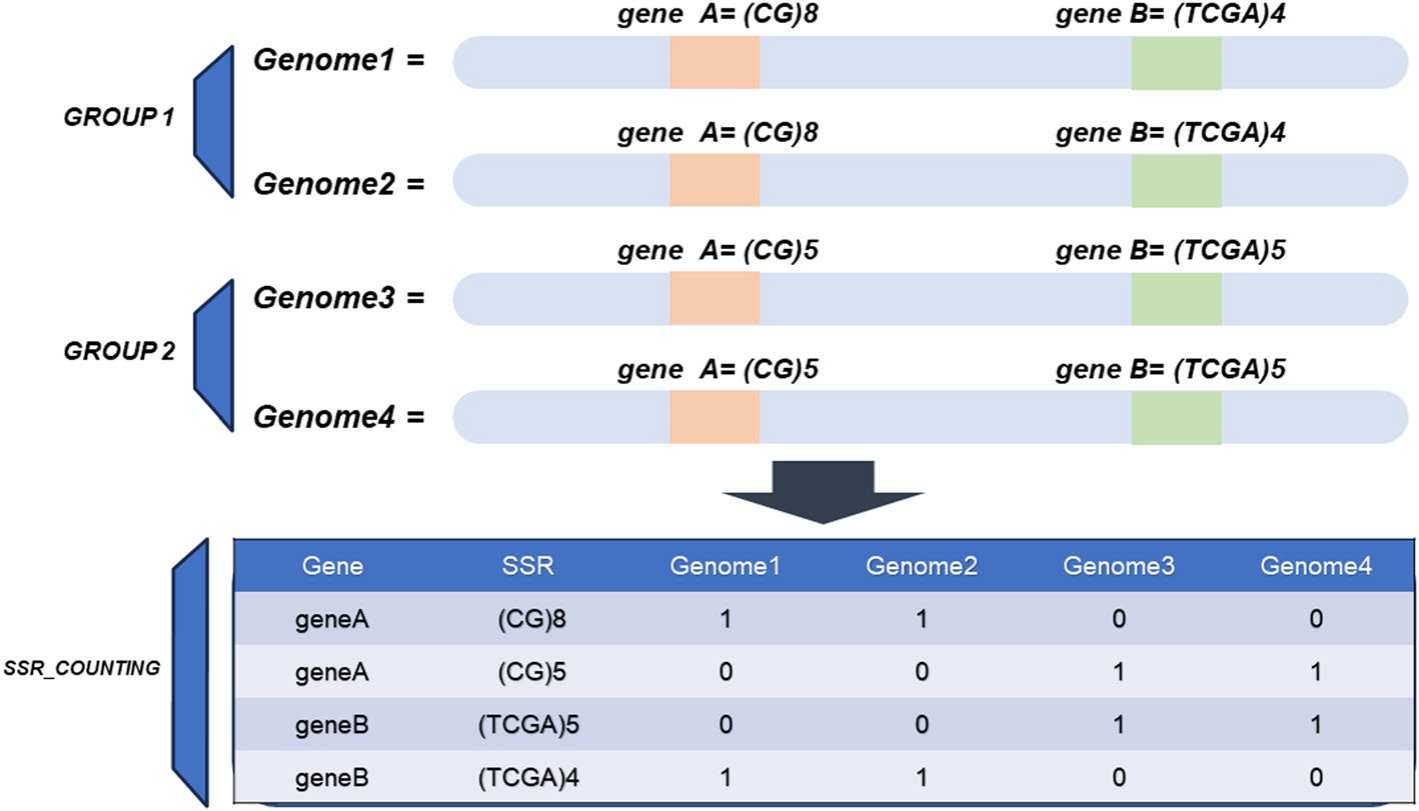
Diagram illustrating the process of counting the SSR in coding regions to identify potential SSR markers after the SSRs mining. The figure depicts the abundance calculation process, where the SSRs are counted on a per-gene basis. Subsequently, the output is analyzed by an R-script that compares the selected SSR among the previously selected groups (Group1 compared to Group2 in the example above)

Additionally, for GenBank data analysis, the obtained results can be further analyzed, by generating a table of microsatellite motif abundance from the identified genic regions in the GenBank file formats of different genomes “SSR_counting.txt” (Fig. 2). This analysis is useful for identifying unique and common motifs in the genic regions of the analyzed genomes for genetic diversity analyses based on the filtered SSRs that show the highest potential as genetic markers. Additionally, another script is available to isolate the sequences of the selected SSRs with their flanking regions into a multifasta file from the results.

We chose to create SATIN as a tool that combines enhanced effectiveness, a distinct methodology, and unique functionalities while adhering to parameters and an output format reminiscent of the MISA software [12], which is one of the most widely used tool [11]. The SATIN’s parameters related to SSR search are stored in the "Parameters.ini" file, which specifies the motifs and the minimum number of motif repetitions (motif: repetition), as well as the minimum size of random sequences to identify some imperfect microsatellites (int parameter).

SATIN generates an output indicating the type of SSRs motif followed by the number of repetitions, the start and end positions of the identified Satellite repetition, and the sequence ID.

### Input file formats

When using FASTA files as input, SATIN can perform the search for Satellites repetitions in two ways: (1) Single Analysis: analyses unique fasta or multifasta file through a command; or (2) Batch analysis of multiple fasta files within a single folder through a unique command.

On the other hand, when GenBank files are used as input, besides generating the same output as in the analysis of FASTA files, SATIN will generate additional output files. (1) A fasta file containing the genome sequence; (2) A Protein Table file that extracts the coding regions; and (3) an SSR-coding file with identified SSRs specifically found within the coding regions. The SSR-coding file is presented in the format of Table 3.

**Table 3:** Example of a file Format displaying SSRs in coding regions, along with gene name and their positions within the coding region

Start	End	SSR	Gene	Strand	Synonym (locus_tag)	Product	ID
583,756	583,763	(A)8	appY	+	b0564	DNA-binding transcriptional activator	NC_000913.3

### Analysis of SSRs in coding regions among groups of genomes

**Fig. 3:**
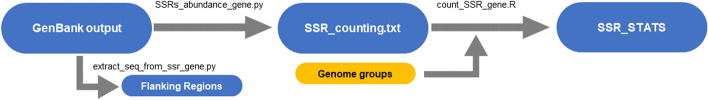
Flowchart of the steps for the perfect SSR analysis of the coding regions among different groups of genomes. The first step is shown in Fig. 3 where the SSR on a per gene basis is counted and saved on a file called “SSR_couting.txt” (abundance file), then is analyzed together with a grouping file by an Rscript to generate results with some statistical analysis such as tests for normality (Shapiro–Wilk), non-parametric Kruskal–Wallis test, parametric ANOVA, Tukey's post hoc test, and a sum of the SSR counts for each gene and SSR. After the SSR has been selected the user can select the flanking regions using a script called “extract_seq_from_ssr_gene.py”

In addition to its main function of SSR mining, SATIN can also serve as a complementary tool by providing scripts for additional analysis of SSRs profiles among pre-defined groups of genomes. These tools will compare and analyze perfect SSRs present among the groups of genomes. The idea is based on the assumption that differences in SSR counts occur due to mutations within these repetitive sequences [20] and that the SSRs diverging among the groups will have their motif count values modified due to population polymorphism [3]. As a result, it generates a file containing the SSRs count values within each gene from a Python script (abundance file) and compares them with the counts present in the other group of genomes under the same conditions using an R script to compare the groups (Figs. 2 and 3).

The tool takes as input the outputs generated by running GenBank format files, specifically, the previously obtained results from SATIN for the coding regions, along with a TSV file defining the group membership of each organism for statistical analysis purposes.

Afterward, SATIN’s coding SSR file results can be used to generate an abundance file that compares the SSR frequency profiles of coding SSRs among each group by executing a Python script, and, subsequently, a count of SSRs within the gene regions is performed for each analyzed genome and saved on an abundance file (Fig. 2). It utilizes the obtained results and segregates them according to the genome files used.

**Fig. 4:**
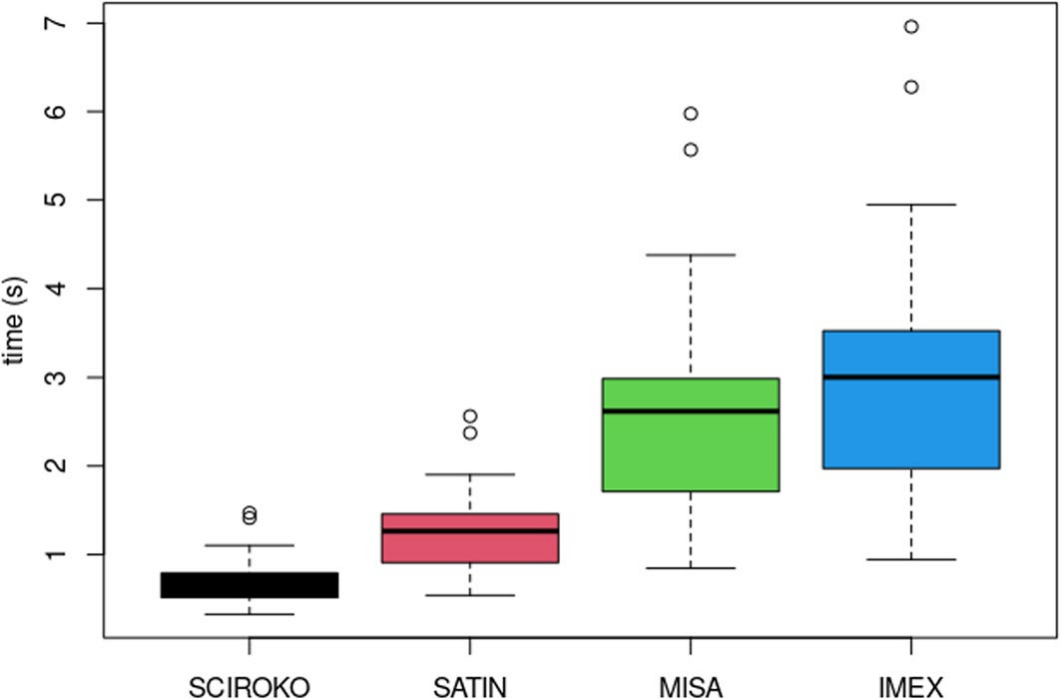
Box plot of the processing time for each of 100 genomes with detection of SSRs under the same parameters. The circles above each box plot represent outliers

Using the abundance file and the pre-defined groups of genomes file, the user can run an R-script to compare these SSRs and make further analyses of differential SSR frequency between groups by using tests for normality (Shapiro–Wilk), non-parametric Kruskal–Wallis test, parametric ANOVA, Tukey's post hoc test, and a sum of the SSR counts for each gene and SSR. The results of these tests are saved in separate files, so the user can analyze the results to select potential SSR markers (Fig. 4).

If the SSRs have already been selected and the user needs to design new primers and therefore needs the flanking regions to do so, SATIN offers a script to extract the flanking regions of each SSR of the selected coding regions present on the folder with all genomes analyzed (Fig. 3). These flanking regions can be used as input in subsequent steps for primer selection using other tools such as GSP [7], ConsensusPrimer [21], or Primer3 [22].

In addition to microsatellite mining, SATIN can utilize its results generated from the identified SSRs in coding regions to compare the SSRs identified by each genome group. It utilizes the obtained results and segregates them according to the genome groups predefined by the user.

The analysis assumes that the SSRs diverging among the groups will have their motif count values modified due to population polymorphism [3]. As a result, it generates a file containing the SSR count values within each gene and compares them with the counts present in other genomes under the same conditions (Fig. 2).

Based on the table generated through the abundance calculation of SSRs, along with an additional file provided by the user indicating the analyzed genomes and their corresponding groups, SATIN offers an R script that analyses the abundance table and groups to identify differentially expressed SSRs among the analyzed groups.

## Results and discussion

### Tools comparison

To compare whether SATIN can generate similar results to other microsatellite mining tools, we used 100 randomly selected genomes from NCBI (supplementary_data1). The motifs of the sequences found by each tool were compared among them as well as the processing time for each task (Fig. 4).

Among the known microsatellite mining programs, the following programs were selected for result and processing time comparison: IMEX-2.1 [13], MISA [12, 18], and Sciroko [23]. Also, we specifically focused on searching for perfect microsatellites to facilitate the analysis of outputs.

**Fig. 5:**
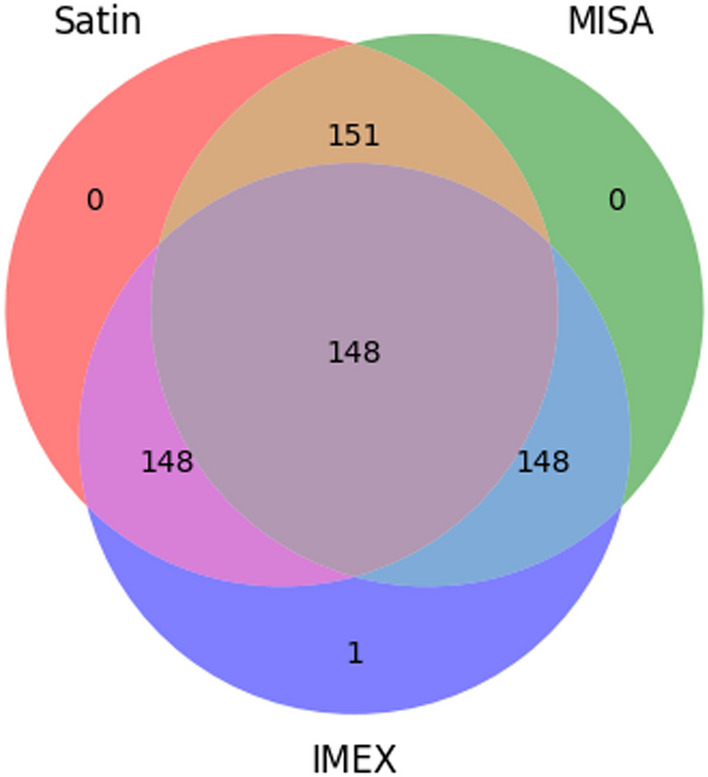
Venn diagram comparing the shared or unique SSR (motif) regions based on the output generated by the programs under the same search conditions. The value at the center indicates the number of motifs identified in common by all three software programs. The three values immediately following, in light blue, brown, and purple, indicate the motifs shared between MISA-IMEX, MISA-SATIN, and IMEX-SATIN, respectively. The remaining values represent the motifs uniquely identified by each software: Green—uniquely identified by MISA, Navy blue—uniquely identified by IMEX, and Red—uniquely identified by SATIN

The motif sizes and repetitions used for the adjusted programs were 1:12, 2:5, 3:4, 4:3, 5:3, and 6:3 based on parameters previously defined [19, 24], where the values correspond to the motif size followed by the minimum number of repetitions. The programs were executed, and their processing time outputs were analyzed as shown (Figs. 4 and 5).

For the comparison of their motifs and identified start and end positions, multiple Venn diagrams were generated for each analyzed genome comparing each of these categories (SSR, start position, and end position). These diagrams compared the number of results common to all three tools for each genome (supplementary data and Fig. 5).

### Processing time

With the previously mentioned data, we also ran SATIN with 3 more programs on MISA mode for 100 genomes and compared their running time for each mining software used.

Based on the data from Fig. 4, it can be observed that SATIN is faster compared to MISA and IMEX-2.1, except for Sciroko, which is known to be a fast SSR mining tool [11]. It is important to note that IMEX-2.1 was built in the same C language as SATIN, while MISA was written in Perl, and this will probably influence the time of processing besides the parameters used for each tool and alternative outputs created.

### Efficiency in microsatellite identification

For the comparison of their motifs, multiple Venn diagrams were generated for each analyzed genome comparing the identified SSRs. These diagrams compared the number of results of perfect SSRs common to three tools (MISA, IMEX, and SATIN) for each genome (Fig. 5 & supplementary_data1). Sciroko's output was not compared due to his differentiated output (not in table format) when compared to MISA and IMEX-2.1.

Figure 5 (below) depicts one of these diagrams for the motifs identified in the *Escherichia coli* str. K-122 genome (GCF_000005845.2), where the common motifs among the three programs are shown at the center, the motifs shared between two of the three software programs are indicated in light blue, brown, and purple colors, and the unique motifs identified for each analyzed program are represented in green, navy blue, and red colors for MISA, IMEX, and SATIN software, respectively.

From the data in Fig. 5, it can be observed that SATIN can identify a similar number of outputs when compared to IMEX and MISA under the same conditions. The SSRs “(GGCG)3”, “(TTAT)3” and “(GGT)4” were only identified by SATIN and MISA. IMEX showed a unique SSR “(GTG)4”, but when closely looked at, “(GTG)4” corresponded to the same SSR “(GGT)4”, so they were considered the same. Giving MISA and SATIN a plus of 2 SSRs. We also ran 100 other genomes with the same conditions to create other Venn diagrams to show this characteristic of SATIN compared to other tools with perfect SSR mining (supplementary_data1).

### Analysis of SSRs for the identification of potential population markers

To test the results of the identified SSRs of the coding regions among populations, the microsatellite data of *Escherichia coli* identified and experimentally validated between the k12 and O157 groups in coding regions were used [25] (Table 4). The identified SSRs in the coding regions in Table 4 were then compared with the SSRs identified by SATIN.

**Table 4:** List of identified SSRs in coding regions among *Escherichia coli* groups K12 and the O157 group. These SSRs were subsequently analyzed with the results obtained from SATIN for result comparison

Strain and substrain	No of repeats	Motif	Genomic location, name of ORF	Source
k12:w3110, b sr9b, ehec, epec, etec	8	C	G1787051, gsiA	[25]
k12:w3110, b sr9b, ehec, epec, etec	9	A	G1790021, yibA	[25]
k12:w3110, b sr9b, ehec, epec, etec	6	GC	G1786541, mhpR	[25]
k12, b, ehec, epec, etec, e:1–69	5	CGG	G1786284, ftsZ	[25]
k12, b, ehec, epec, etec, e:1–69	4	CTGG	G1788332, hisC	(25)

For the identification of SSRs, 51 genomes of *Escherichia coli* strains from the K12 group and 51 genomes of *Escherichia coli* strains from the O157 group were used (supplementary_data2). These data were downloaded from NCBI (RefSeq) in GenBank format and underwent microsatellite mining by SATIN using the following parameters: 1:8, 2:6, 3:4, 4:3, 5:3, and 6:3; defined [19, 24] and adapted based on Table 4. The identified SSRs in gene regions were then subjected to abundance calculation, resulting in the "SSR_counting.txt" file, which was later analyzed using R.

As a result, since the data did not show a normal distribution from Shapiro–Wilk results, non-parametric tests were considered. Among the identified SSRs for these groups, approximately 237 SSRs were found in gene regions with a p-value less than 0.001 in the Kruskal–Wallis analysis (supplementary_data2). Among these identified SSRs, 5 SSRs mentioned in Table 4 were identified (Table 5).

**Table 5:** Association of identified SSRs in the same genes listed in Table 4 between *Escherichia coli* groups of the K12 and O157 strains using SATIN. Note that the gene b0829 was identified by the name “gsiA”

SSR	Gene	AOV	AOV	AOV	AOV	AOV	SHAPIRO–WILK	SHAPIRO–WILK	KRUSKAL–WALLIS	KRUSKAL–WALLIS
		Pr(> F)	F_value	Mean_Sq	Sum_Sq	Df	W	P-value	chi_squared	p_value
(A)9	yibA	1.45 × 10^–72^	2500	24.51	24.51	1	0.6363	1.57 × 10^–14^	1	6.54 × 10^–23^
(C)8	gsiA	2.66 × 10^–11^	56.25	7147	7147	1	0.5503	3.71 × 10^–16^	1	1.64 × 10^–09^
(CGG)5	ftsZ	4.10 × 10^–10^	48.08	6127	6127	1	0.5341	1.94 × 10^–16^	1	1.03 × 10^–08^
(GC)6	mhpR	1.43 × 10^–06^	26.31	4324	4324	1	0.5649	6.73 × 10^–16^	1	4.50 × 10^–06 ^
(GCTG)4	hisC	1.08 × 10^–10^	52	6627	6627	1	0.5424	2.70 × 10^–16^	1	4.15 × 10^–09^

*AOV, Analysis of variance

From the table with Kruskal–Wallis results available on supplementary_data2, we also selected other 2 SSRs (one per gene), that were probably differentiated from the elongation/shortening process, from the 237 genes to show how these markers can differentiate between the two serovars (K12 and O157 groups) of *Escherichia coli*. These 2 SSRs had their flanking regions extracted and individually analyzed to see how they would differentiate among the two serovars.

The first SSR identified were the (CGG)4 and (CGG)5 repeats in the *accC* gene. The SSR (CGG)4 were present on the genomes of the O157 serovar and (CGG)5 were present on the genomes of the K12 serovar. The second SSR identified was the (A)8 and (A)9 repeats in the *yqeJ* gene that showed to be repeated twice in that region. In the genomes with the K12 serovar, it was observed the repetition (A)8 twice in the *yqeJ* gene [(A)8, (A)8], while in the O157 serovar, it was observed the repetition (A)8 and (A)9 in the *yqeJ* gene [(A)8, (A)9]. However, one of the K12 genomes (GCF_003028735.1) showed a (CGG)4 repetition in the *accC* gene and not all O157 genomes could be identified due to this gene being absent on some of the GenBank files for both SSRs (supplementary_data2).

In summary, SATIN provides an output similar to MISA, and faster processing time compared to other programs and enables additional analyses of potential population markers for microsatellites. It can analyze the frequency of specific SSRs to distinct population groups and generate suggestions of SSRs that exhibit differential frequency among them based on some statistical analysis. If desired, the user can isolate these SSR regions for further population-based analyses, particularly focusing on the loci associated with the SSRs.

Besides Minisatellites being not discussed on the results, SATIN’s algorithm can identify bigger motif sizes when defined by the user, since it produces a numerical list defined by that motif size. So, the SATIN motif is limited only by the hardware where it’s been used.

## Conclusion

In conclusion, SATIN emerges as a valuable and efficient tool for the detection of mini and microsatellites using a new algorithm based on prime numbers, as it can identify a wide range of SSRs present in genomes. SATIN also displays some additional tools to help identify new markers, such as the identification of the SSRs present in the coding regions and further statistical analysis to help the user to better identify perfect repeats polymorphism in coding regions. Its ability to analyze different types of SSRs and its fast processing make it a versatile and practical choice for researchers working with SSR analysis.

## Availability and requirements

Availability and Implementation: SATIN is available at https://github.com/labgm/SATIN.git.

Project name: SATIN—Micro and Mini SATellite IdentificatioN tool.

Project home page: https://github.com/labgm/SATIN.git

Operating system(s): Linux.

Programming language: C, Python, and R.

Other requirements: pandas, numpy, biopython, collection, libz-dev, libbz2-dev, r-base (‘dplyr’, ‘tidyr’).

License: MIT License.

Any restrictions to use by non-acad: none.

### Supplementary Information

Supplementary file 1

Supplementary file 2

## Data Availability

All data generated or analyzed during this study are included in this published article in the “Supplementary_Files”. The genomes used in Supplementary_Files 1 and 2 can be downloaded from NCBI through GenBank accession number or via the FTP links available on the “data_and_materials.txt” files.

## Abbreviations

SSR: Simple sequence repeat
SATIN: Micro and Mini SATellite IdentificatioN tool
TRs: Tandem repeats
SRs: Satellite repeats
TSV: Tab separated values
ANOVA: AOV analysis of variance
