# Supplementary figures and images for: SATIN: a micro and mini satellite mining tool of total genome and coding regions with analysis of perfect repeats polymorphism in coding regions (part 2 of 2)
Source: BMC Bioinformatics. 2024 Jun 18;25:217. doi: 10.1186/s12859-024-05842-2 (PMC11186120; doi:10.1186/s12859-024-05842-2)

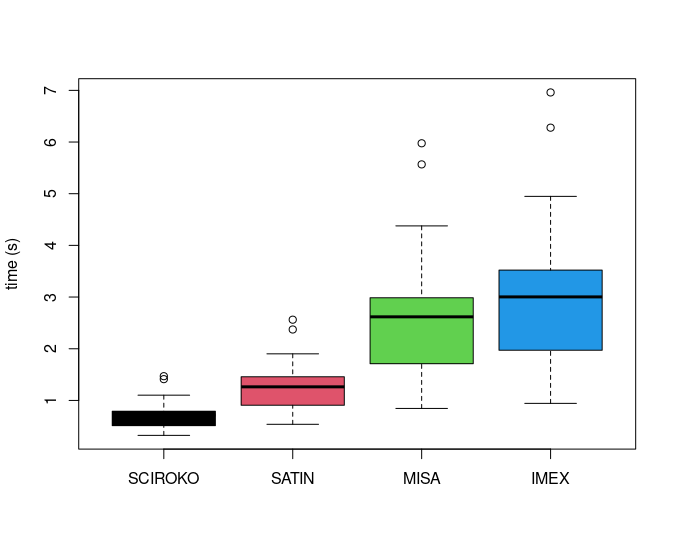

Supplement: Supplementary file 1 [file 12859_2024_5842_MOESM1_ESM.zip › Supplementary File1/time/fig4.png]
