# Supplementary figures and images for: SATIN: a micro and mini satellite mining tool of total genome and coding regions with analysis of perfect repeats polymorphism in coding regions (part 1 of 2)
Source: BMC Bioinformatics. 2024 Jun 18;25:217. doi: 10.1186/s12859-024-05842-2 (PMC11186120; doi:10.1186/s12859-024-05842-2)

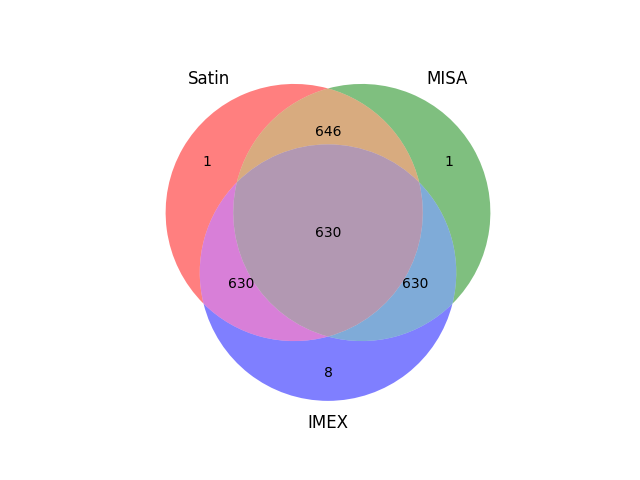

Supplement: Supplementary file 1 [file 12859_2024_5842_MOESM1_ESM.zip › Supplementary File1/SSR_venn_diagram/GCF_000026445.2_ASM2644v2_genomic.fna_SSR.png]

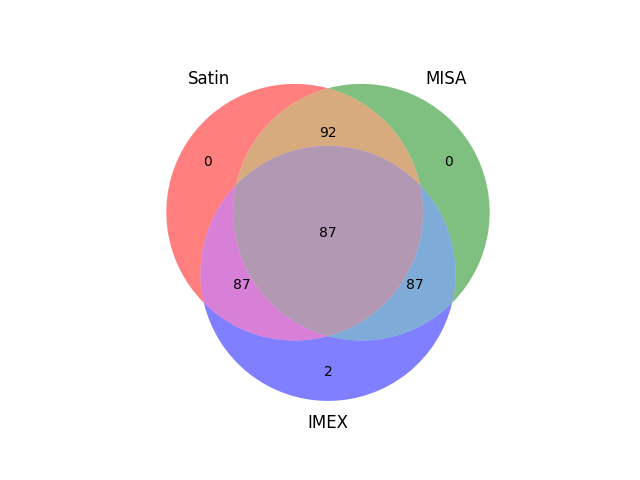

Supplement: Supplementary file 1 [file 12859_2024_5842_MOESM1_ESM.zip › Supplementary File1/SSR_venn_diagram/GCF_000253695.1_ASM25369v2_genomic.fna_SSR.png]

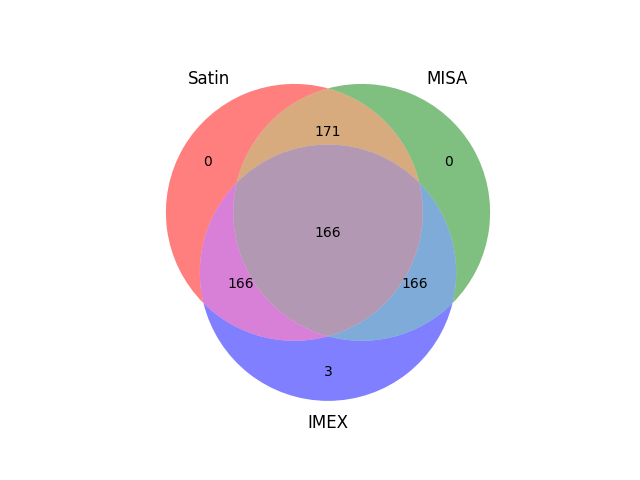

Supplement: Supplementary file 1 [file 12859_2024_5842_MOESM1_ESM.zip › Supplementary File1/SSR_venn_diagram/GCF_000263995.1_ASM26399v1_genomic.fna_SSR.png]

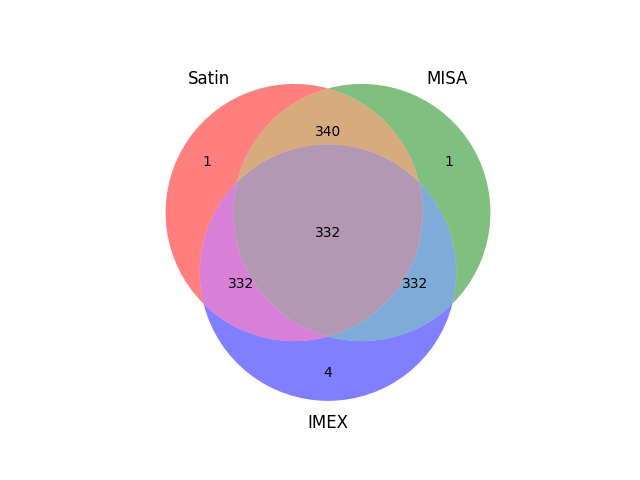

Supplement: Supplementary file 1 [file 12859_2024_5842_MOESM1_ESM.zip › Supplementary File1/SSR_venn_diagram/GCF_000316625.1_ASM31662v1_genomic.fna_SSR.png]

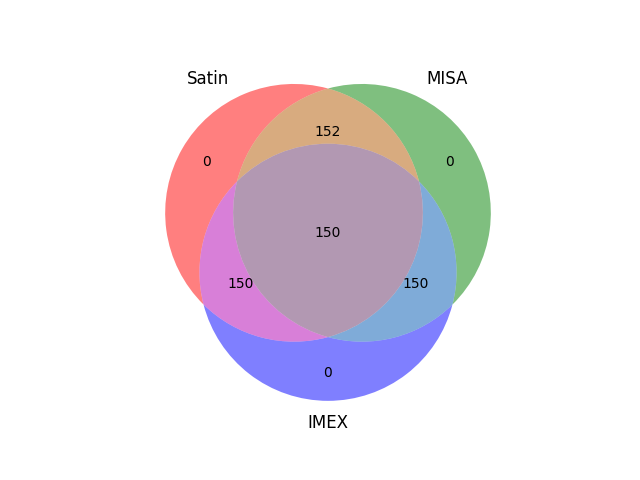

Supplement: Supplementary file 1 [file 12859_2024_5842_MOESM1_ESM.zip › Supplementary File1/SSR_venn_diagram/GCF_000326665.1_Esch_coli_KTE99_V1_genomic.fna_SSR.png]

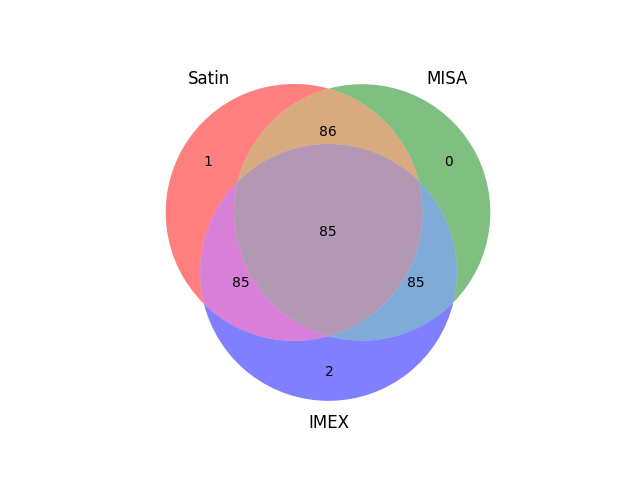

Supplement: Supplementary file 1 [file 12859_2024_5842_MOESM1_ESM.zip › Supplementary File1/SSR_venn_diagram/GCF_000331025.1_ASM33102v1_genomic.fna_SSR.png]

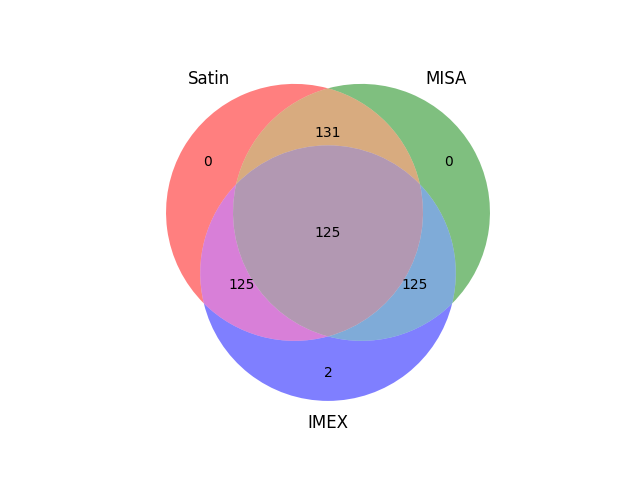

Supplement: Supplementary file 1 [file 12859_2024_5842_MOESM1_ESM.zip › Supplementary File1/SSR_venn_diagram/GCF_000589655.1_ASM58965v1_genomic.fna_SSR.png]

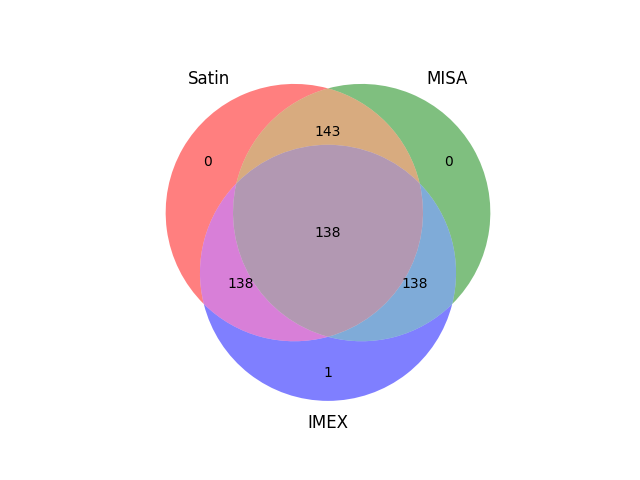

Supplement: Supplementary file 1 [file 12859_2024_5842_MOESM1_ESM.zip › Supplementary File1/SSR_venn_diagram/GCF_000596685.1_Stap_aure_H32126_V1_genomic.fna_SSR.png]

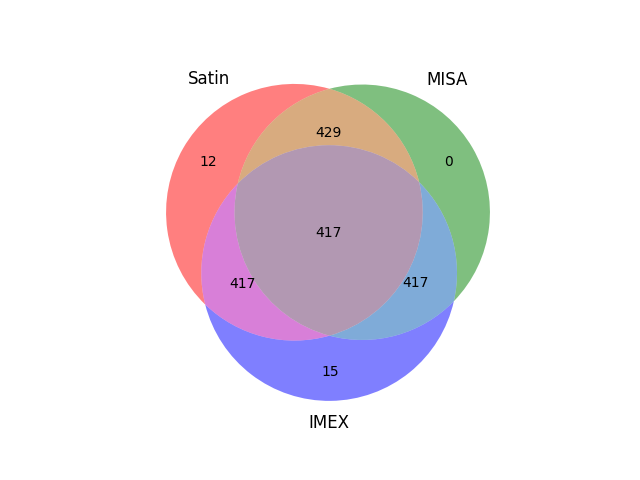

Supplement: Supplementary file 1 [file 12859_2024_5842_MOESM1_ESM.zip › Supplementary File1/SSR_venn_diagram/GCF_000655655.1_Myco_tube_XTB13-214_V1_genomic.fna_SSR.png]

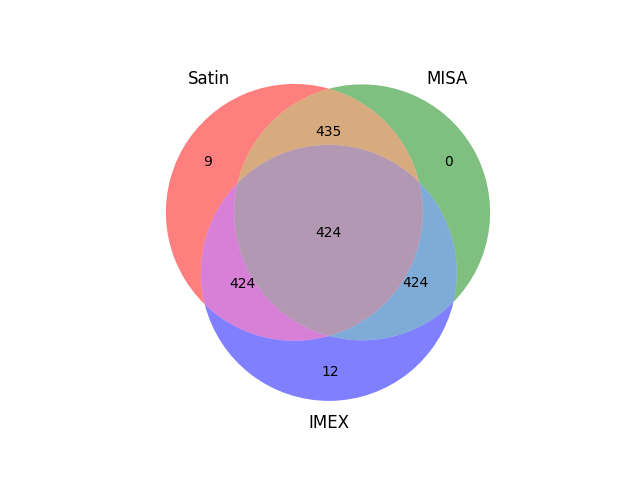

Supplement: Supplementary file 1 [file 12859_2024_5842_MOESM1_ESM.zip › Supplementary File1/SSR_venn_diagram/GCF_000662605.1_Myco_tube_M1340_V1_genomic.fna_SSR.png]

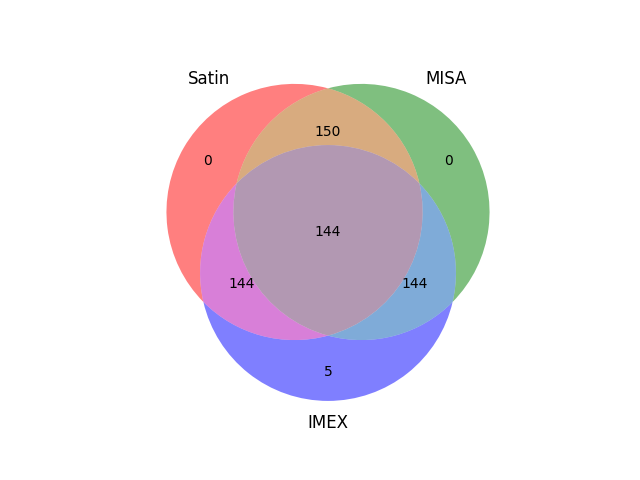

Supplement: Supplementary file 1 [file 12859_2024_5842_MOESM1_ESM.zip › Supplementary File1/SSR_venn_diagram/GCF_000735135.1_ASM73513v1_genomic.fna_SSR.png]

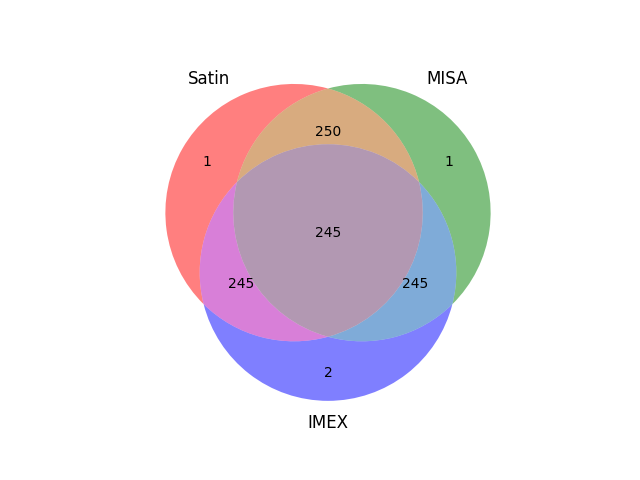

Supplement: Supplementary file 1 [file 12859_2024_5842_MOESM1_ESM.zip › Supplementary File1/SSR_venn_diagram/GCF_000957835.1_ASM95783v1_genomic.fna_SSR.png]

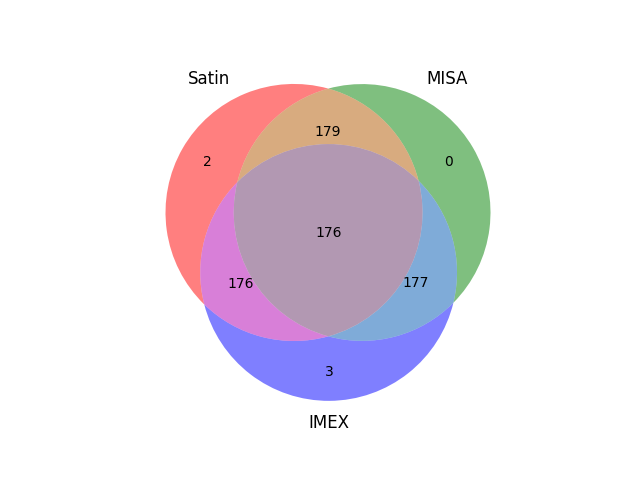

Supplement: Supplementary file 1 [file 12859_2024_5842_MOESM1_ESM.zip › Supplementary File1/SSR_venn_diagram/GCF_001012545.1_CFSAN026844_01.0_genomic.fna_SSR.png]

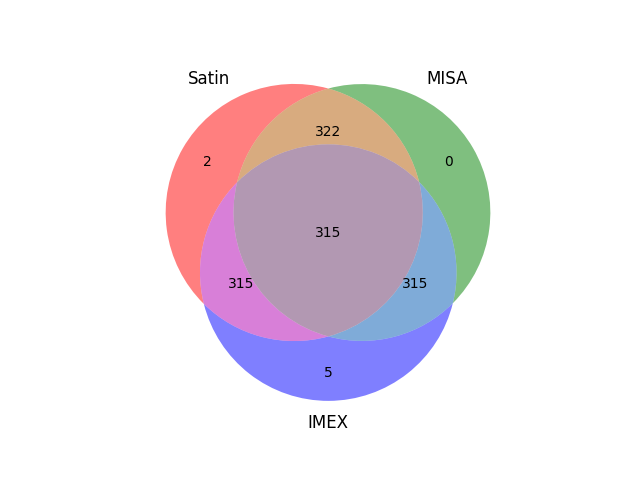

Supplement: Supplementary file 1 [file 12859_2024_5842_MOESM1_ESM.zip › Supplementary File1/SSR_venn_diagram/GCF_001052565.1_ASM105256v1_genomic.fna_SSR.png]

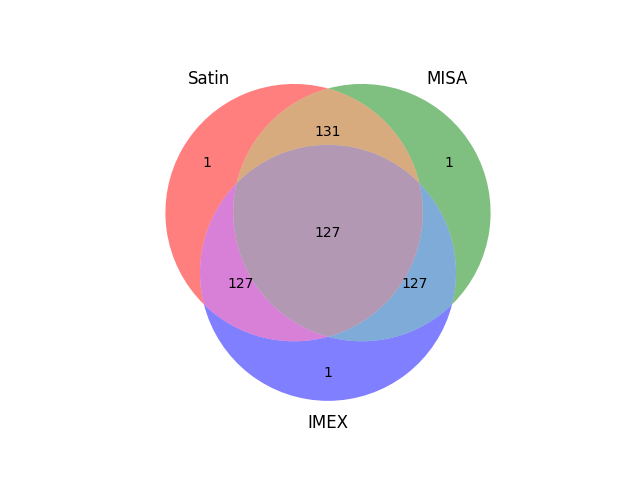

Supplement: Supplementary file 1 [file 12859_2024_5842_MOESM1_ESM.zip › Supplementary File1/SSR_venn_diagram/GCF_001060985.1_ASM106098v1_genomic.fna_SSR.png]

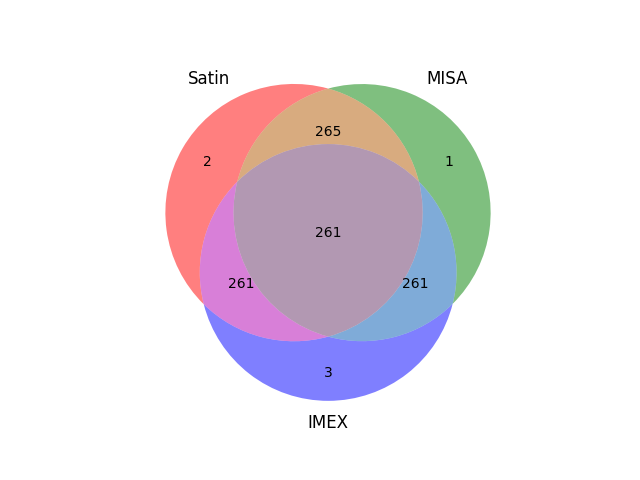

Supplement: Supplementary file 1 [file 12859_2024_5842_MOESM1_ESM.zip › Supplementary File1/SSR_venn_diagram/GCF_001134405.1_9870_8_88_genomic.fna_SSR.png]

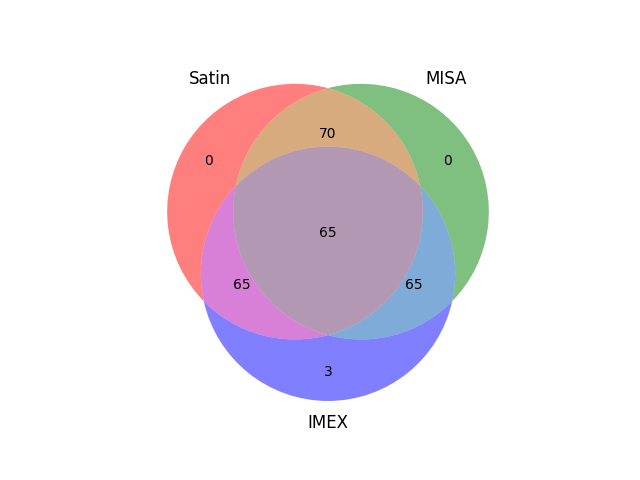

Supplement: Supplementary file 1 [file 12859_2024_5842_MOESM1_ESM.zip › Supplementary File1/SSR_venn_diagram/GCF_001135725.1_7054_6_13_genomic.fna_SSR.png]

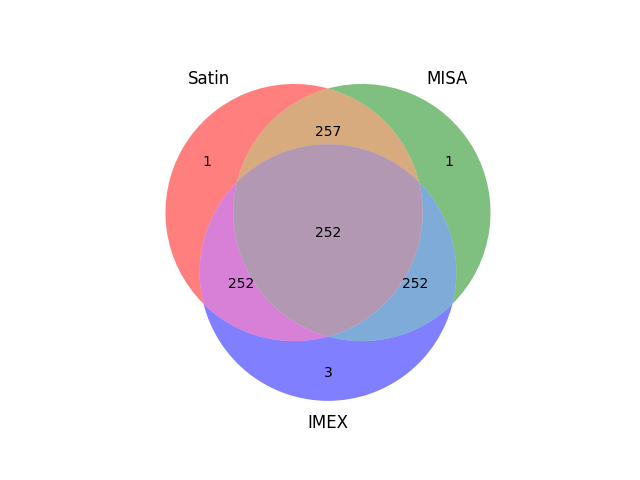

Supplement: Supplementary file 1 [file 12859_2024_5842_MOESM1_ESM.zip › Supplementary File1/SSR_venn_diagram/GCF_001168365.1_10592_2_55_genomic.fna_SSR.png]

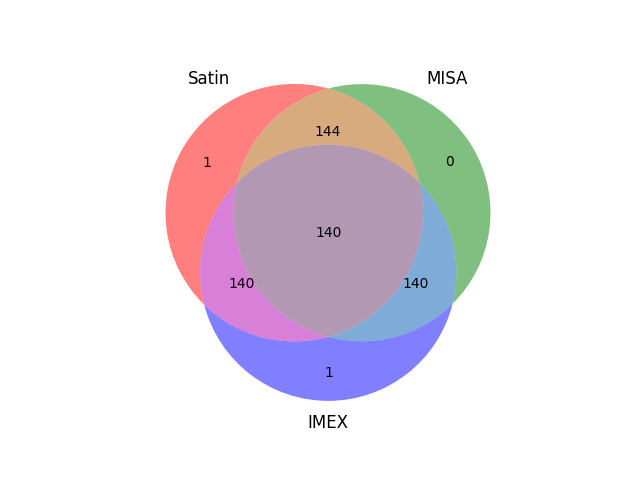

Supplement: Supplementary file 1 [file 12859_2024_5842_MOESM1_ESM.zip › Supplementary File1/SSR_venn_diagram/GCF_001233145.1_6401_7_9_genomic.fna_SSR.png]

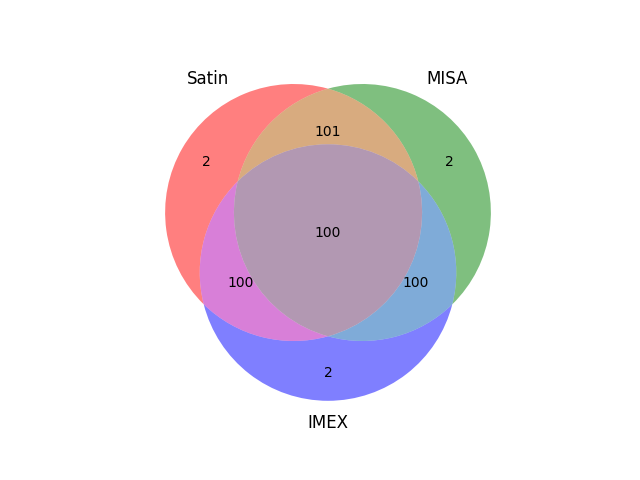

Supplement: Supplementary file 1 [file 12859_2024_5842_MOESM1_ESM.zip › Supplementary File1/SSR_venn_diagram/GCF_001236645.1_7213_3_38_genomic.fna_SSR.png]

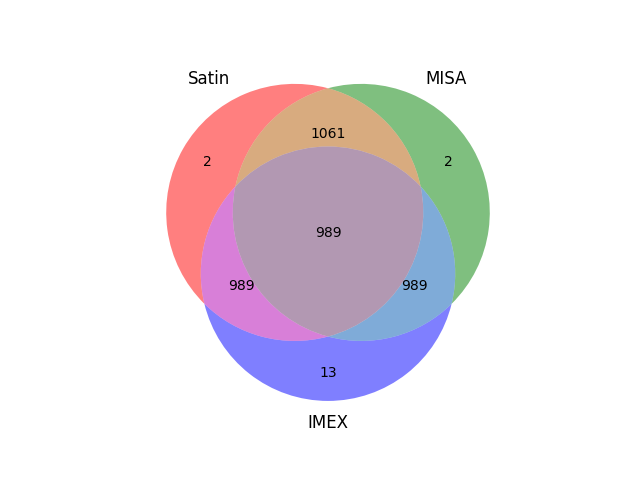

Supplement: Supplementary file 1 [file 12859_2024_5842_MOESM1_ESM.zip › Supplementary File1/SSR_venn_diagram/GCF_001279345.1_ASM127934v1_genomic.fna_SSR.png]

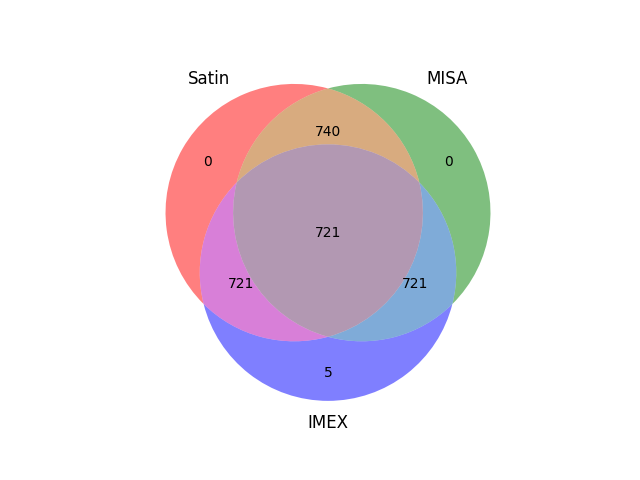

Supplement: Supplementary file 1 [file 12859_2024_5842_MOESM1_ESM.zip › Supplementary File1/SSR_venn_diagram/GCF_001307415.1_ASM130741v1_genomic.fna_SSR.png]

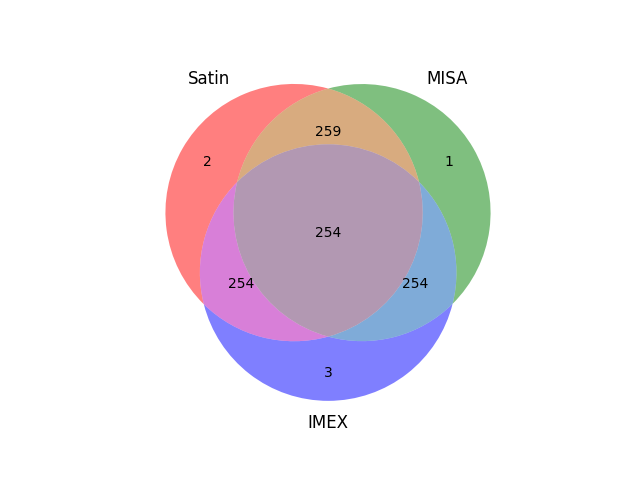

Supplement: Supplementary file 1 [file 12859_2024_5842_MOESM1_ESM.zip › Supplementary File1/SSR_venn_diagram/GCF_001368515.1_10607_2_41_genomic.fna_SSR.png]

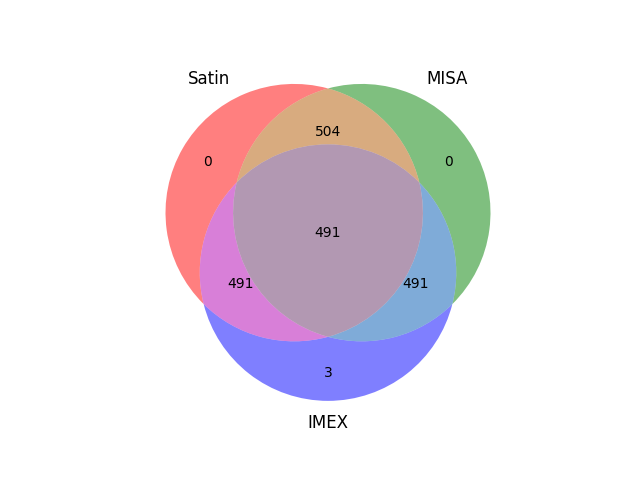

Supplement: Supplementary file 1 [file 12859_2024_5842_MOESM1_ESM.zip › Supplementary File1/SSR_venn_diagram/GCF_001373055.1_E204_genomic.fna_SSR.png]

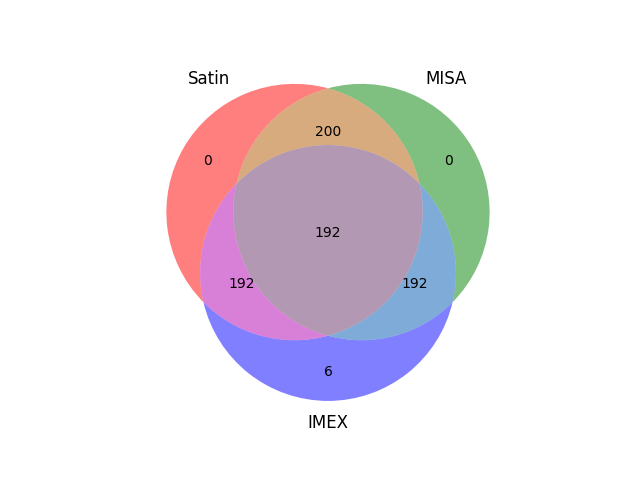

Supplement: Supplementary file 1 [file 12859_2024_5842_MOESM1_ESM.zip › Supplementary File1/SSR_venn_diagram/GCF_001412215.1_ASM141221v1_genomic.fna_SSR.png]

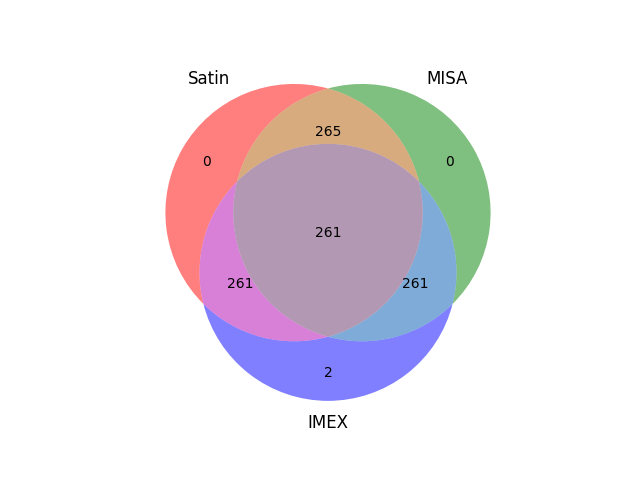

Supplement: Supplementary file 1 [file 12859_2024_5842_MOESM1_ESM.zip › Supplementary File1/SSR_venn_diagram/GCF_001481035.1_Salmonella_enterica_CVM_N42232_v1.0_genomic.fna_SSR.png]

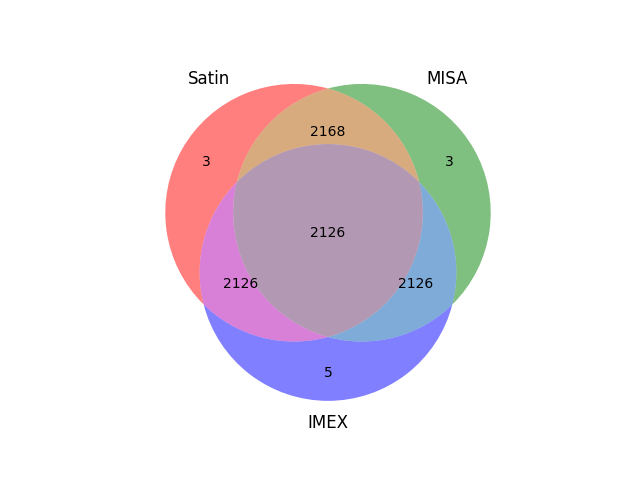

Supplement: Supplementary file 1 [file 12859_2024_5842_MOESM1_ESM.zip › Supplementary File1/SSR_venn_diagram/GCF_001532815.1_ASM153281v1_genomic.fna_SSR.png]

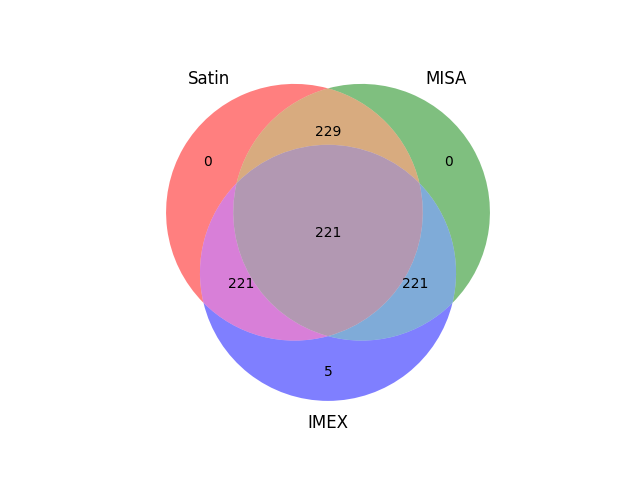

Supplement: Supplementary file 1 [file 12859_2024_5842_MOESM1_ESM.zip › Supplementary File1/SSR_venn_diagram/GCF_001558115.1_ASM155811v1_genomic.fna_SSR.png]

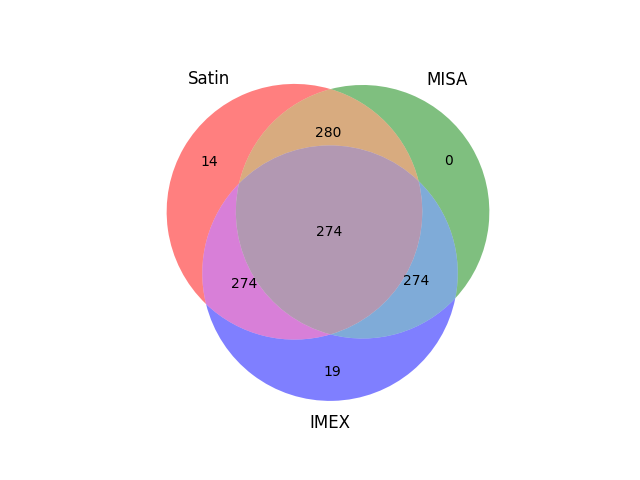

Supplement: Supplementary file 1 [file 12859_2024_5842_MOESM1_ESM.zip › Supplementary File1/SSR_venn_diagram/GCF_001559785.1_ASM155978v1_genomic.fna_SSR.png]

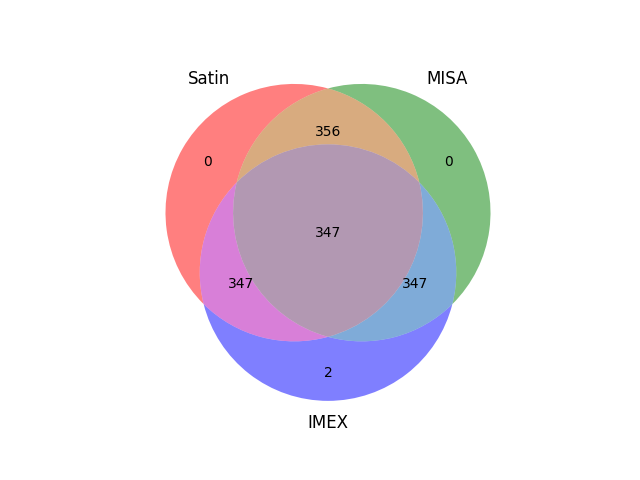

Supplement: Supplementary file 1 [file 12859_2024_5842_MOESM1_ESM.zip › Supplementary File1/SSR_venn_diagram/GCF_001631845.1_ASM163184v1_genomic.fna_SSR.png]

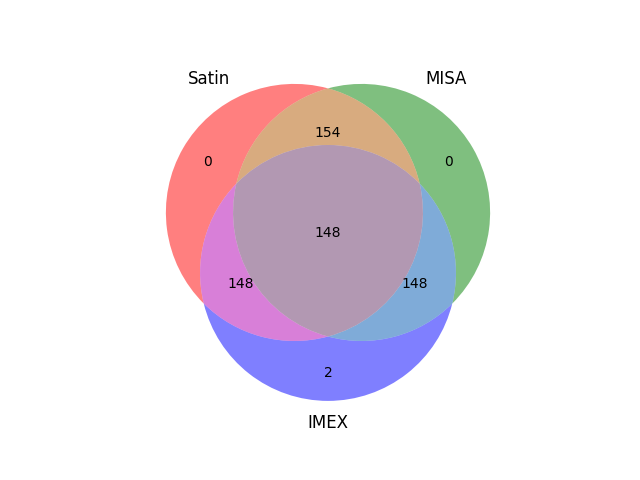

Supplement: Supplementary file 1 [file 12859_2024_5842_MOESM1_ESM.zip › Supplementary File1/SSR_venn_diagram/GCF_001648435.1_ASM164843v1_genomic.fna_SSR.png]

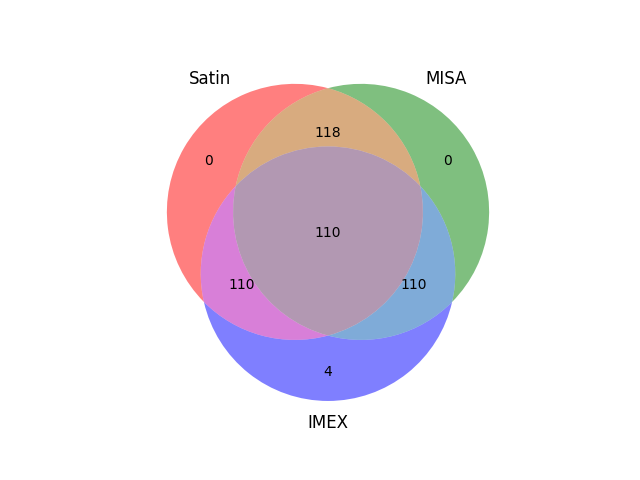

Supplement: Supplementary file 1 [file 12859_2024_5842_MOESM1_ESM.zip › Supplementary File1/SSR_venn_diagram/GCF_001669195.1_ASM166919v1_genomic.fna_SSR.png]

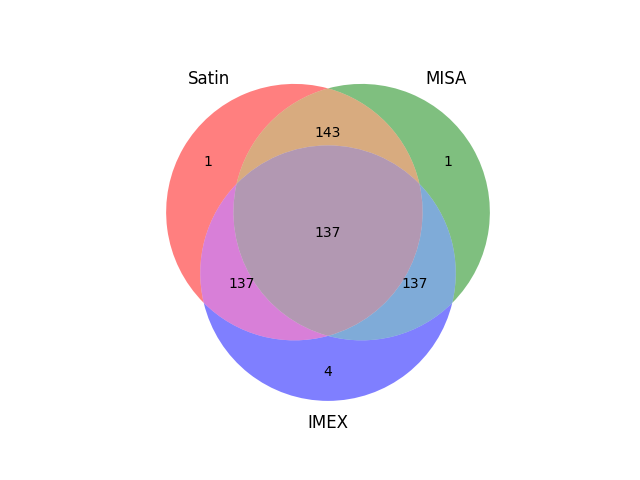

Supplement: Supplementary file 1 [file 12859_2024_5842_MOESM1_ESM.zip › Supplementary File1/SSR_venn_diagram/GCF_001693185.1_ASM169318v1_genomic.fna_SSR.png]

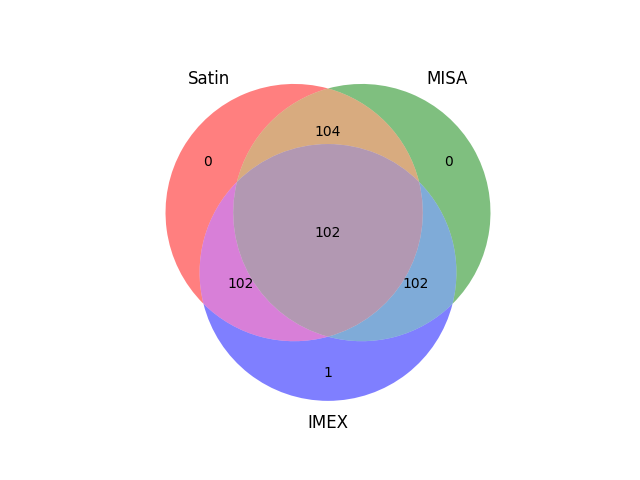

Supplement: Supplementary file 1 [file 12859_2024_5842_MOESM1_ESM.zip › Supplementary File1/SSR_venn_diagram/GCF_001708755.1_ASM170875v1_genomic.fna_SSR.png]

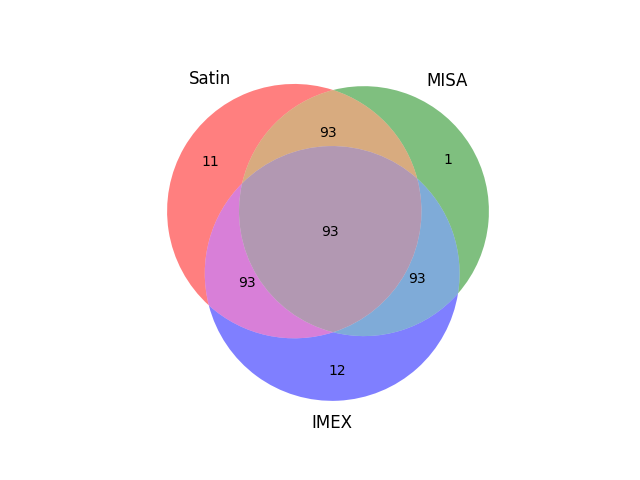

Supplement: Supplementary file 1 [file 12859_2024_5842_MOESM1_ESM.zip › Supplementary File1/SSR_venn_diagram/GCF_001761705.1_ASM176170v1_genomic.fna_SSR.png]

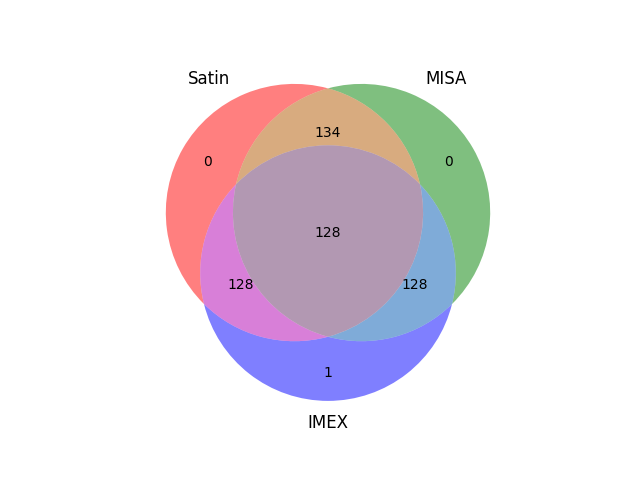

Supplement: Supplementary file 1 [file 12859_2024_5842_MOESM1_ESM.zip › Supplementary File1/SSR_venn_diagram/GCF_001816105.1_ASM181610v1_genomic.fna_SSR.png]

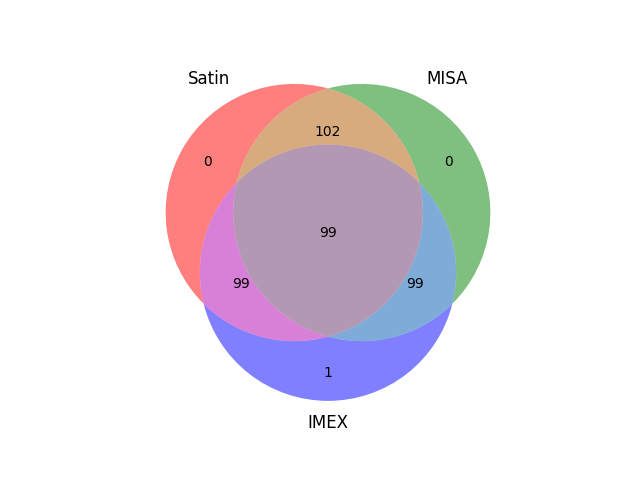

Supplement: Supplementary file 1 [file 12859_2024_5842_MOESM1_ESM.zip › Supplementary File1/SSR_venn_diagram/GCF_001867795.1_ASM186779v1_genomic.fna_SSR.png]

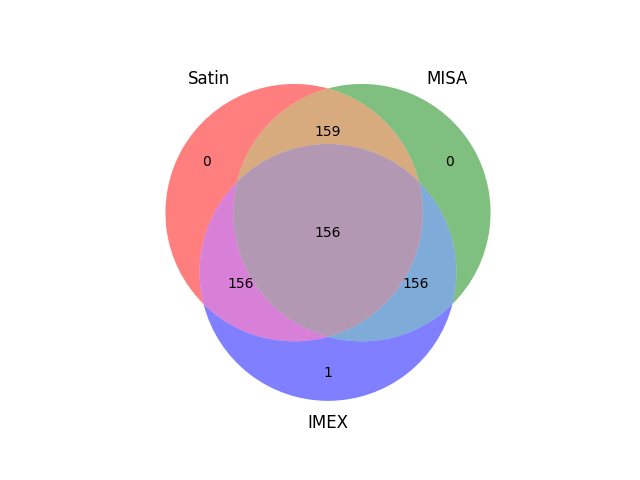

Supplement: Supplementary file 1 [file 12859_2024_5842_MOESM1_ESM.zip › Supplementary File1/SSR_venn_diagram/GCF_002011985.1_ASM201198v1_genomic.fna_SSR.png]

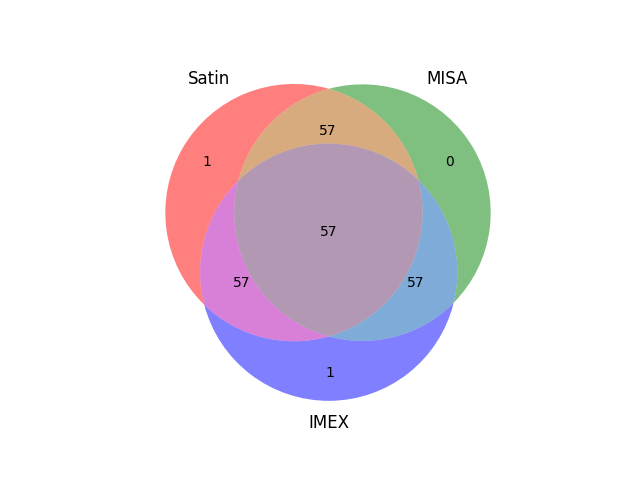

Supplement: Supplementary file 1 [file 12859_2024_5842_MOESM1_ESM.zip › Supplementary File1/SSR_venn_diagram/GCF_002015155.1_ASM201515v1_genomic.fna_SSR.png]

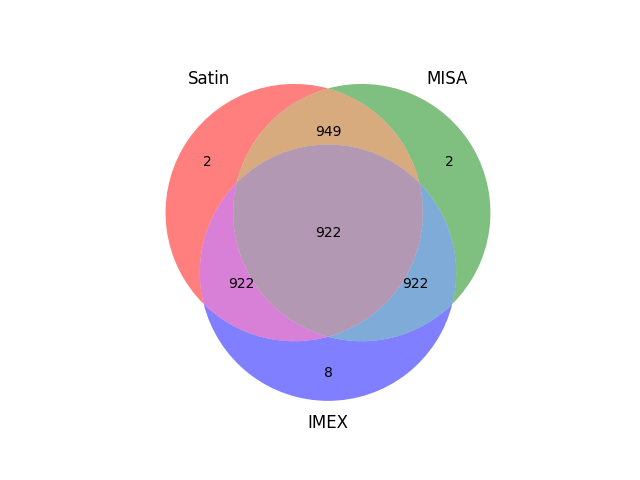

Supplement: Supplementary file 1 [file 12859_2024_5842_MOESM1_ESM.zip › Supplementary File1/SSR_venn_diagram/GCF_002086635.1_ASM208663v1_genomic.fna_SSR.png]

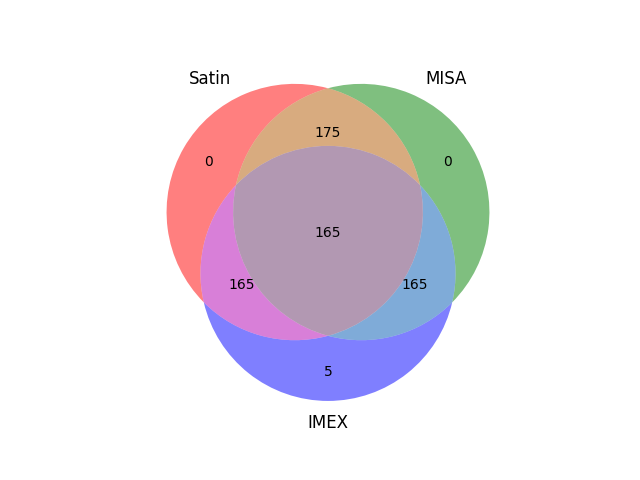

Supplement: Supplementary file 1 [file 12859_2024_5842_MOESM1_ESM.zip › Supplementary File1/SSR_venn_diagram/GCF_002088905.1_ASM208890v1_genomic.fna_SSR.png]

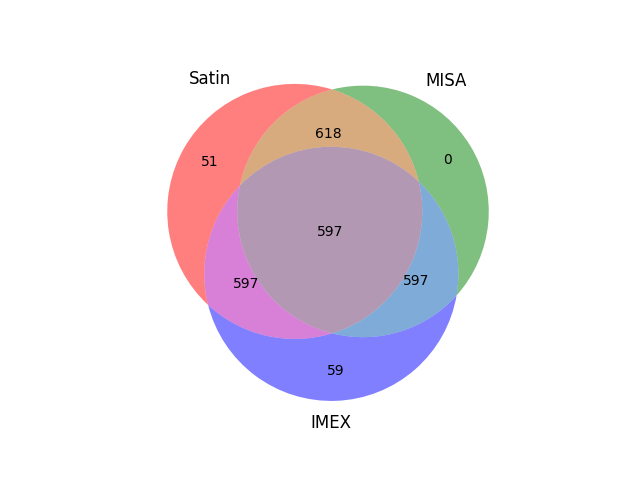

Supplement: Supplementary file 1 [file 12859_2024_5842_MOESM1_ESM.zip › Supplementary File1/SSR_venn_diagram/GCF_002150765.1_ASM215076v1_genomic.fna_SSR.png]

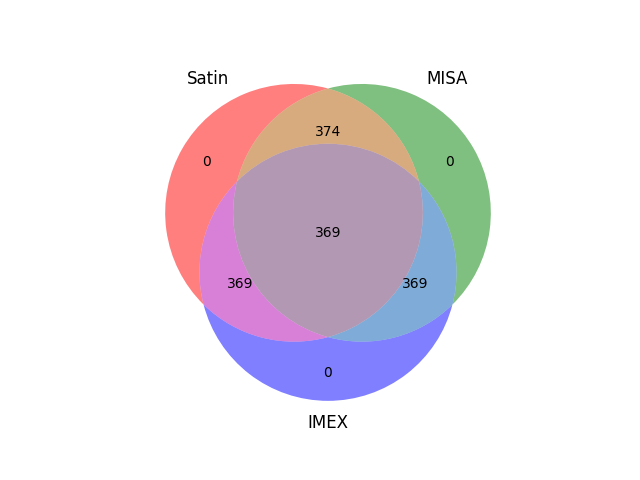

Supplement: Supplementary file 1 [file 12859_2024_5842_MOESM1_ESM.zip › Supplementary File1/SSR_venn_diagram/GCF_002187995.1_ASM218799v1_genomic.fna_SSR.png]

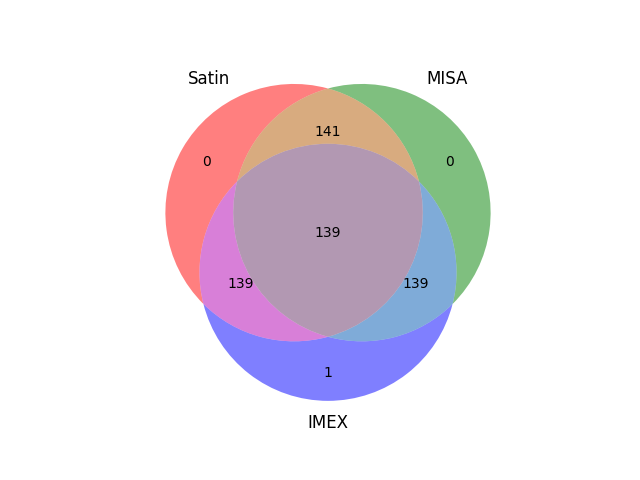

Supplement: Supplementary file 1 [file 12859_2024_5842_MOESM1_ESM.zip › Supplementary File1/SSR_venn_diagram/GCF_002248755.1_ASM224875v1_genomic.fna_SSR.png]

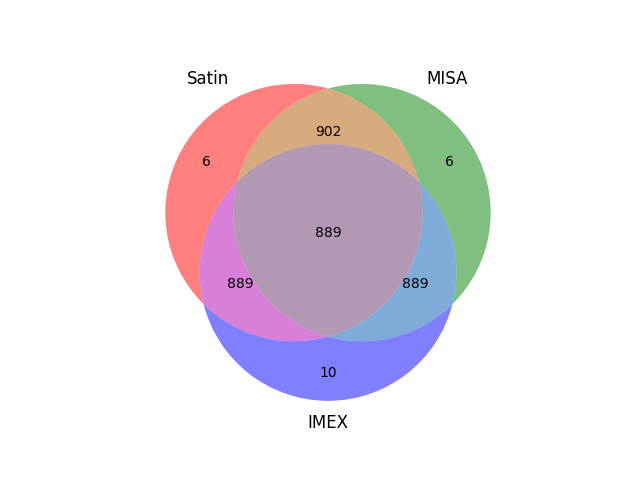

Supplement: Supplementary file 1 [file 12859_2024_5842_MOESM1_ESM.zip › Supplementary File1/SSR_venn_diagram/GCF_002261315.1_ASM226131v1_genomic.fna_SSR.png]

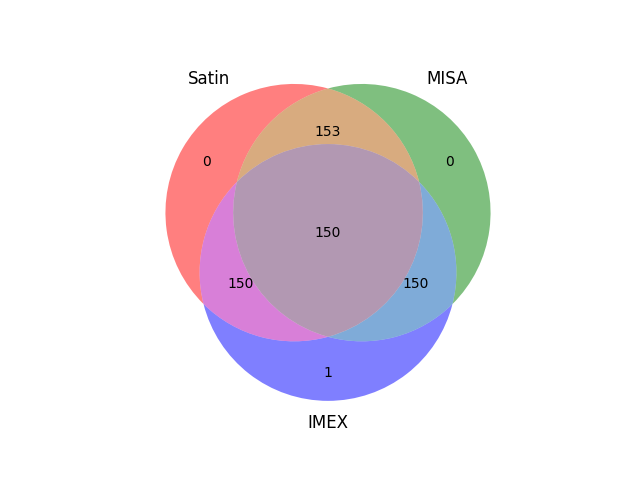

Supplement: Supplementary file 1 [file 12859_2024_5842_MOESM1_ESM.zip › Supplementary File1/SSR_venn_diagram/GCF_002460505.1_ASM246050v1_genomic.fna_SSR.png]

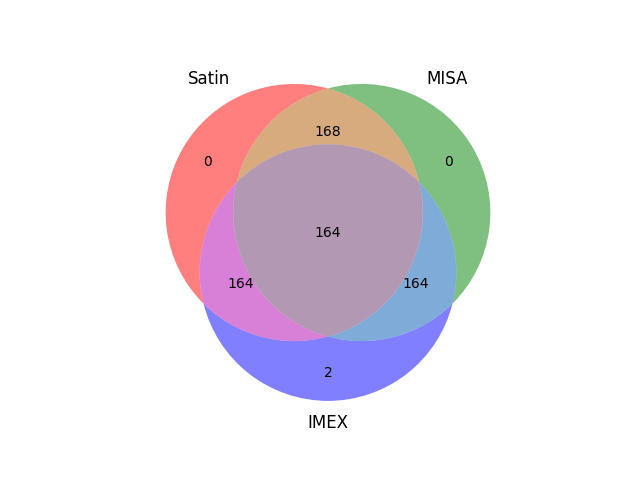

Supplement: Supplementary file 1 [file 12859_2024_5842_MOESM1_ESM.zip › Supplementary File1/SSR_venn_diagram/GCF_002514335.1_ASM251433v1_genomic.fna_SSR.png]

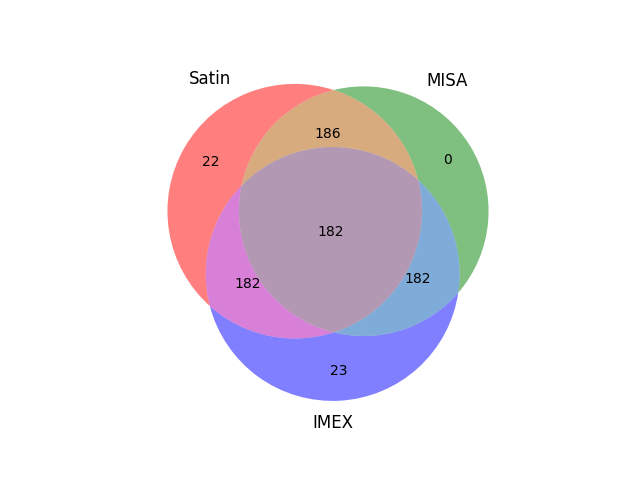

Supplement: Supplementary file 1 [file 12859_2024_5842_MOESM1_ESM.zip › Supplementary File1/SSR_venn_diagram/GCF_002734865.1_ASM273486v1_genomic.fna_SSR.png]

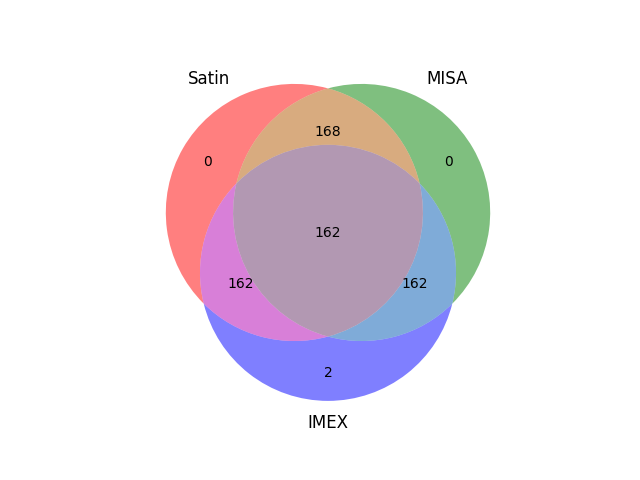

Supplement: Supplementary file 1 [file 12859_2024_5842_MOESM1_ESM.zip › Supplementary File1/SSR_venn_diagram/GCF_003017935.1_ASM301793v1_genomic.fna_SSR.png]

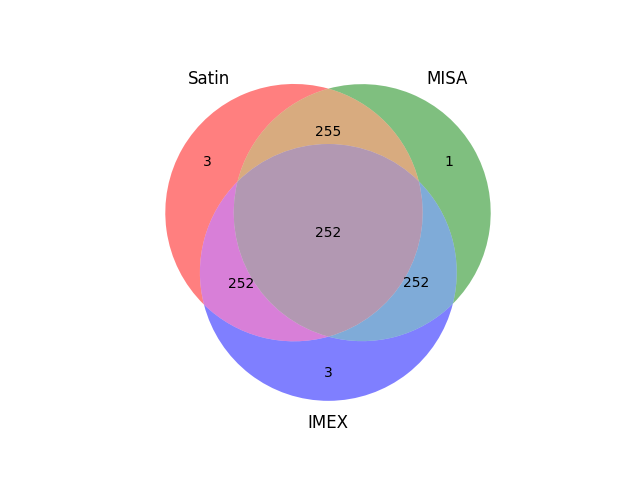

Supplement: Supplementary file 1 [file 12859_2024_5842_MOESM1_ESM.zip › Supplementary File1/SSR_venn_diagram/GCF_003070125.1_ASM307012v1_genomic.fna_SSR.png]

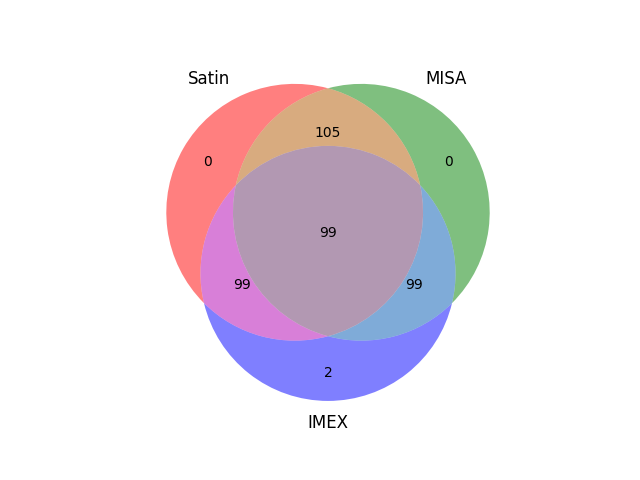

Supplement: Supplementary file 1 [file 12859_2024_5842_MOESM1_ESM.zip › Supplementary File1/SSR_venn_diagram/GCF_003070665.1_ASM307066v1_genomic.fna_SSR.png]

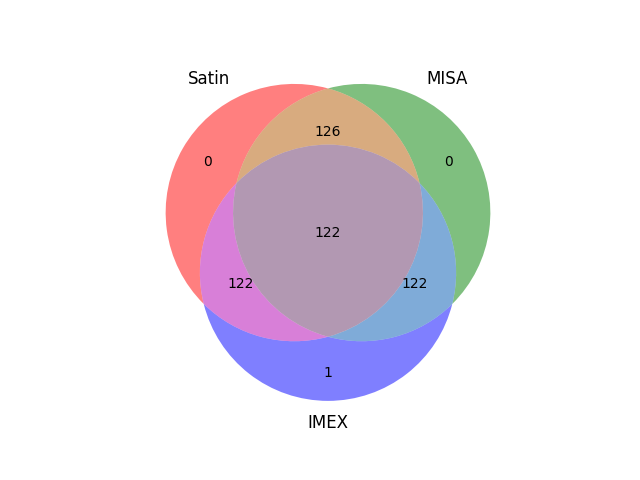

Supplement: Supplementary file 1 [file 12859_2024_5842_MOESM1_ESM.zip › Supplementary File1/SSR_venn_diagram/GCF_003112615.1_ASM311261v1_genomic.fna_SSR.png]

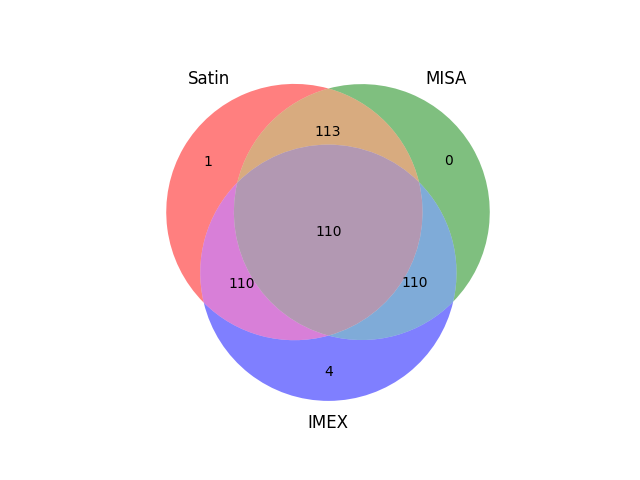

Supplement: Supplementary file 1 [file 12859_2024_5842_MOESM1_ESM.zip › Supplementary File1/SSR_venn_diagram/GCF_003149045.1_ASM314904v1_genomic.fna_SSR.png]

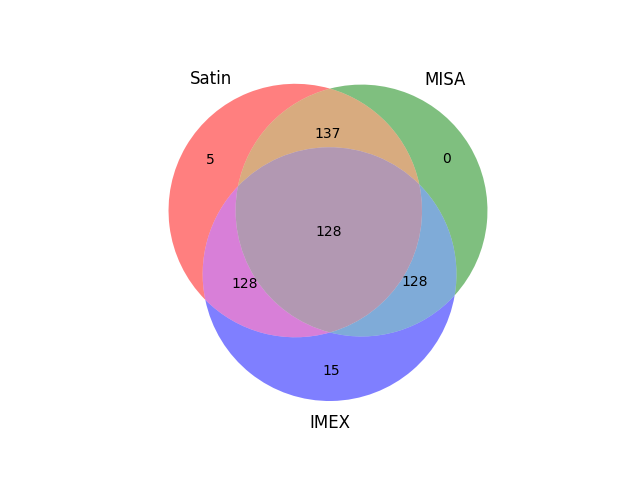

Supplement: Supplementary file 1 [file 12859_2024_5842_MOESM1_ESM.zip › Supplementary File1/SSR_venn_diagram/GCF_003352725.1_ASM335272v1_genomic.fna_SSR.png]

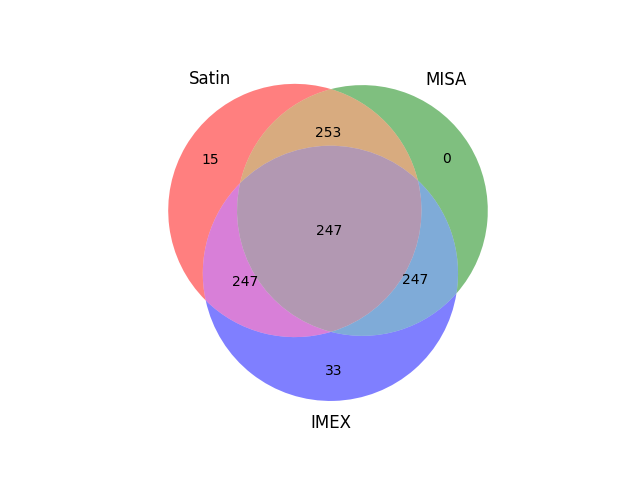

Supplement: Supplementary file 1 [file 12859_2024_5842_MOESM1_ESM.zip › Supplementary File1/SSR_venn_diagram/GCF_003463205.1_ASM346320v1_genomic.fna_SSR.png]

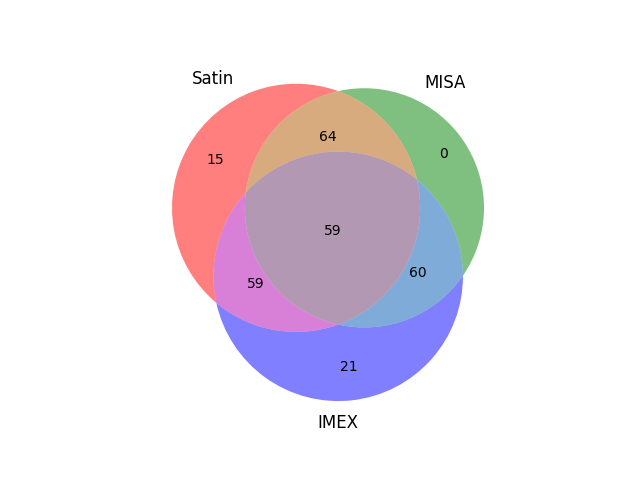

Supplement: Supplementary file 1 [file 12859_2024_5842_MOESM1_ESM.zip › Supplementary File1/SSR_venn_diagram/GCF_003472485.1_ASM347248v1_genomic.fna_SSR.png]

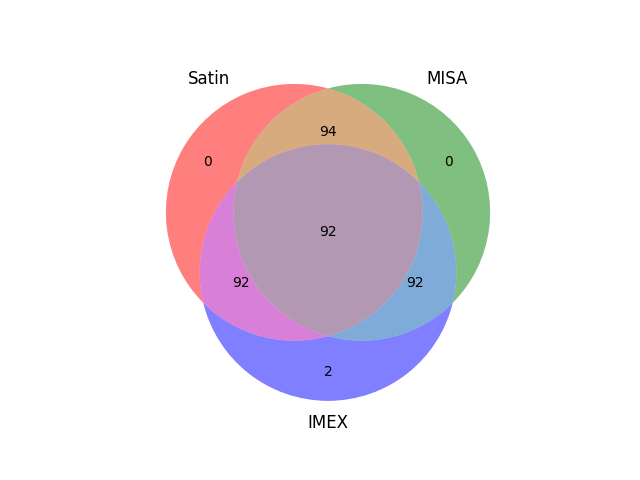

Supplement: Supplementary file 1 [file 12859_2024_5842_MOESM1_ESM.zip › Supplementary File1/SSR_venn_diagram/GCF_003473345.1_ASM347334v1_genomic.fna_SSR.png]

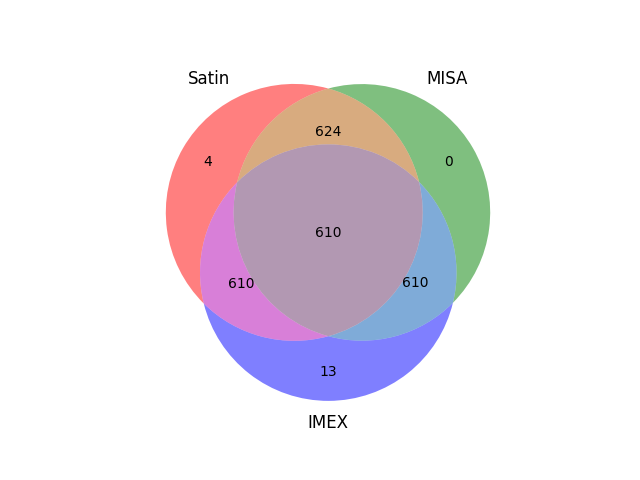

Supplement: Supplementary file 1 [file 12859_2024_5842_MOESM1_ESM.zip › Supplementary File1/SSR_venn_diagram/GCF_003725095.1_ASM372509v1_genomic.fna_SSR.png]

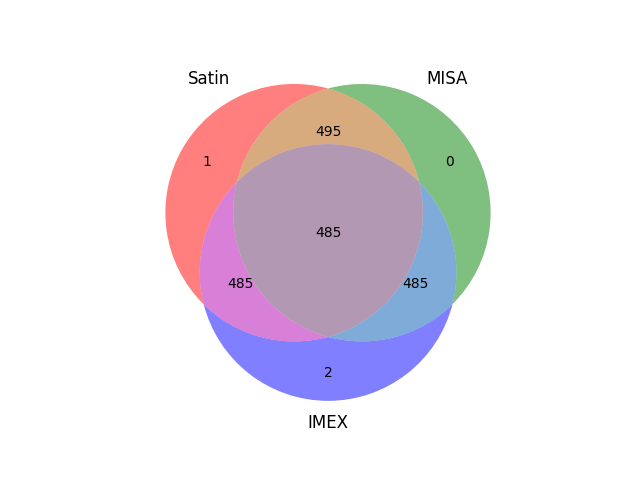

Supplement: Supplementary file 1 [file 12859_2024_5842_MOESM1_ESM.zip › Supplementary File1/SSR_venn_diagram/GCF_003839145.1_ASM383914v1_genomic.fna_SSR.png]

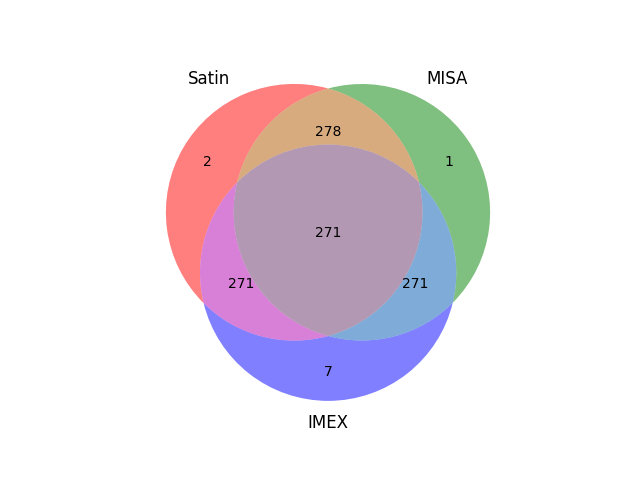

Supplement: Supplementary file 1 [file 12859_2024_5842_MOESM1_ESM.zip › Supplementary File1/SSR_venn_diagram/GCF_003965105.1_ASM396510v1_genomic.fna_SSR.png]

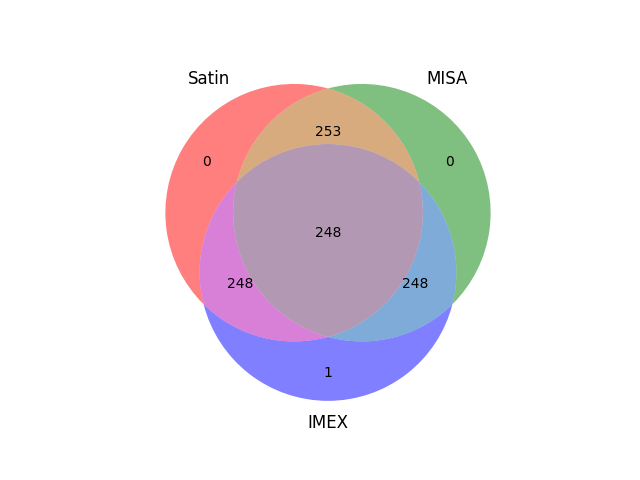

Supplement: Supplementary file 1 [file 12859_2024_5842_MOESM1_ESM.zip › Supplementary File1/SSR_venn_diagram/GCF_004126495.1_ASM412649v1_genomic.fna_SSR.png]

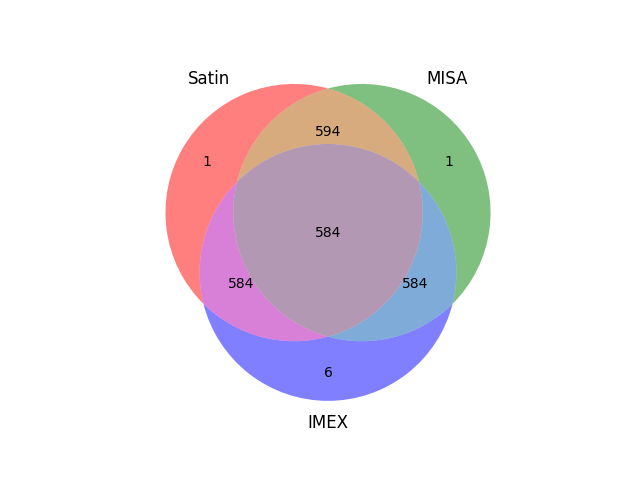

Supplement: Supplementary file 1 [file 12859_2024_5842_MOESM1_ESM.zip › Supplementary File1/SSR_venn_diagram/GCF_004522445.1_ASM452244v1_genomic.fna_SSR.png]

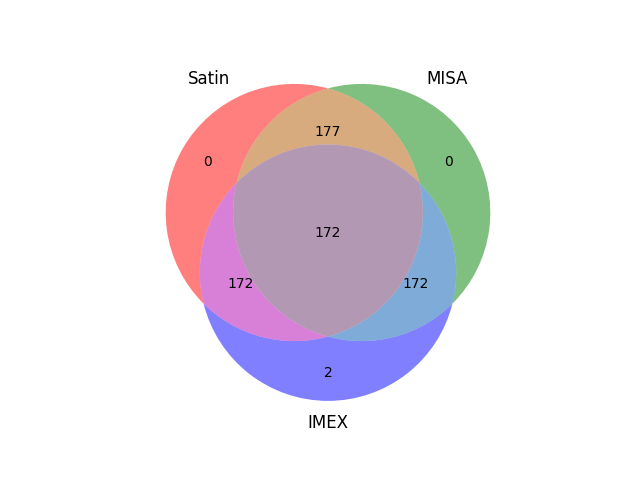

Supplement: Supplementary file 1 [file 12859_2024_5842_MOESM1_ESM.zip › Supplementary File1/SSR_venn_diagram/GCF_005222325.1_ASM522232v1_genomic.fna_SSR.png]

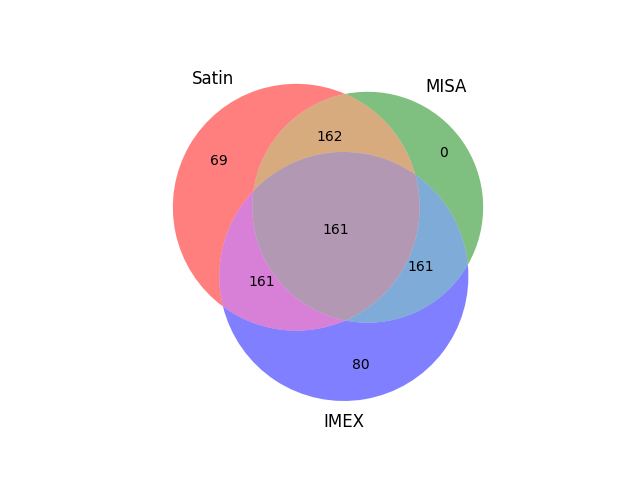

Supplement: Supplementary file 1 [file 12859_2024_5842_MOESM1_ESM.zip › Supplementary File1/SSR_venn_diagram/GCF_005387705.1_ASM538770v1_genomic.fna_SSR.png]

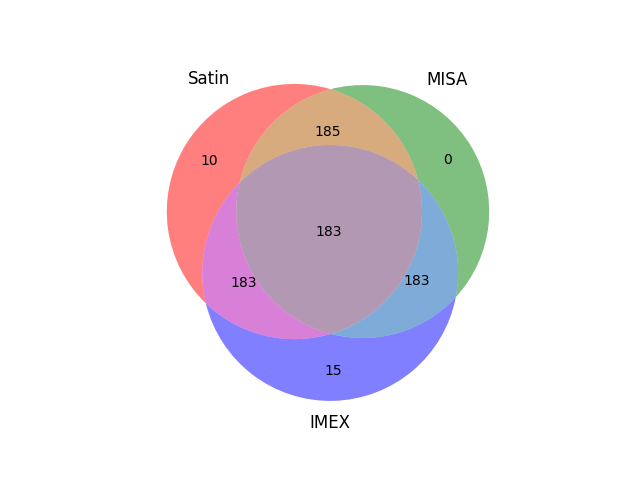

Supplement: Supplementary file 1 [file 12859_2024_5842_MOESM1_ESM.zip › Supplementary File1/SSR_venn_diagram/GCF_005400705.1_ASM540070v1_genomic.fna_SSR.png]

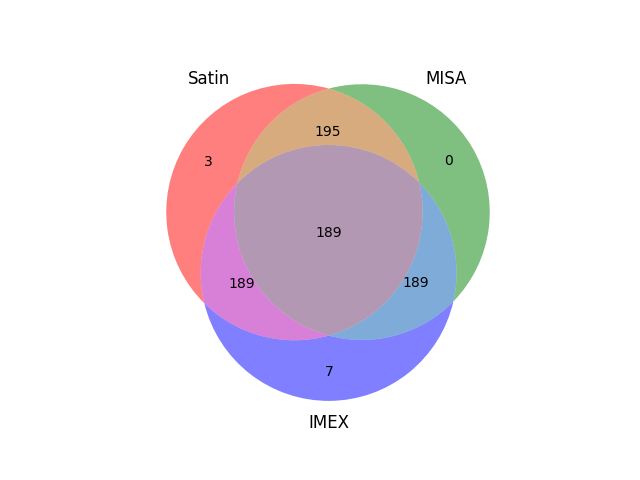

Supplement: Supplementary file 1 [file 12859_2024_5842_MOESM1_ESM.zip › Supplementary File1/SSR_venn_diagram/GCF_005844535.1_ASM584453v1_genomic.fna_SSR.png]

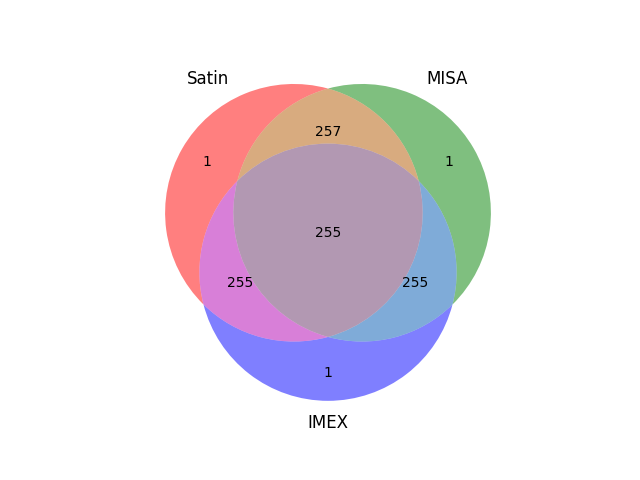

Supplement: Supplementary file 1 [file 12859_2024_5842_MOESM1_ESM.zip › Supplementary File1/SSR_venn_diagram/GCF_006379525.2_ASM637952v2_genomic.fna_SSR.png]

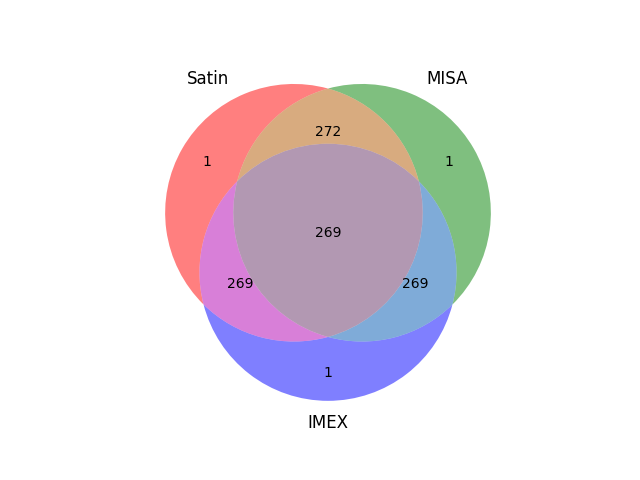

Supplement: Supplementary file 1 [file 12859_2024_5842_MOESM1_ESM.zip › Supplementary File1/SSR_venn_diagram/GCF_006937935.1_ASM693793v1_genomic.fna_SSR.png]

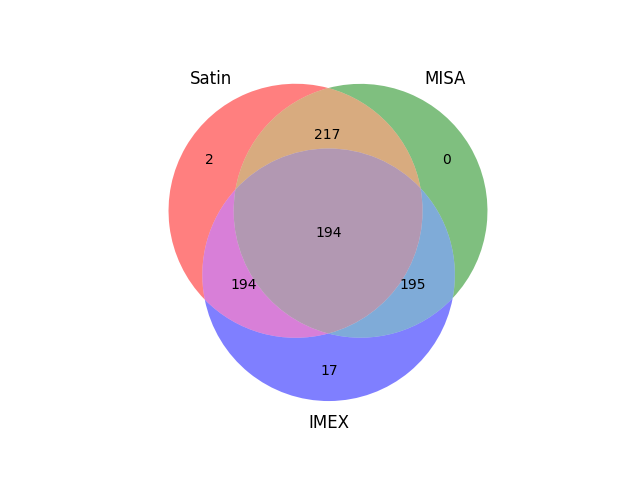

Supplement: Supplementary file 1 [file 12859_2024_5842_MOESM1_ESM.zip › Supplementary File1/SSR_venn_diagram/GCF_007993715.1_ASM799371v1_genomic.fna_SSR.png]

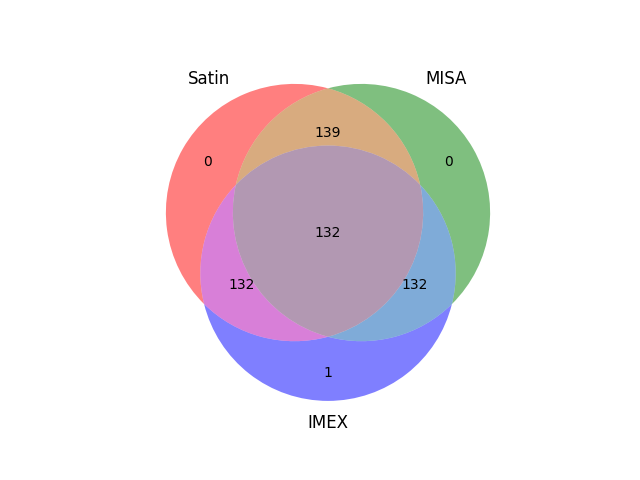

Supplement: Supplementary file 1 [file 12859_2024_5842_MOESM1_ESM.zip › Supplementary File1/SSR_venn_diagram/GCF_008619415.1_ASM861941v1_genomic.fna_SSR.png]

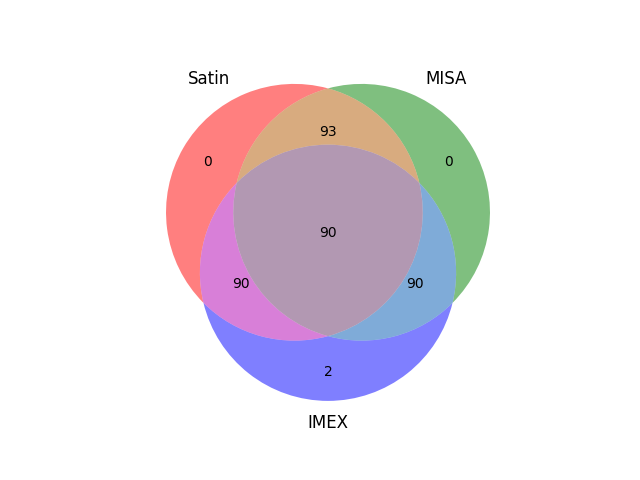

Supplement: Supplementary file 1 [file 12859_2024_5842_MOESM1_ESM.zip › Supplementary File1/SSR_venn_diagram/GCF_008689965.1_ASM868996v1_genomic.fna_SSR.png]

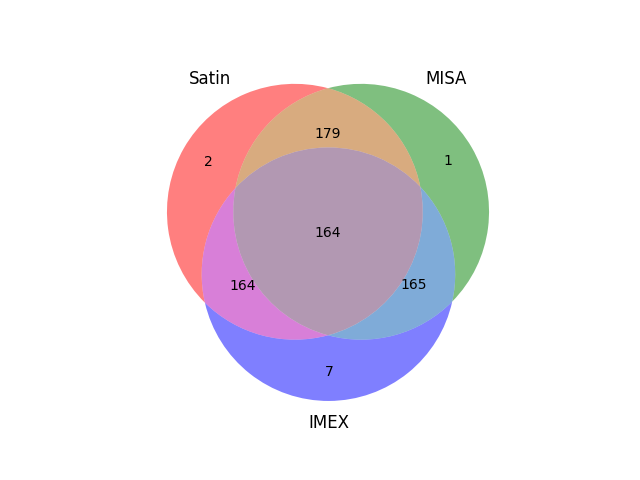

Supplement: Supplementary file 1 [file 12859_2024_5842_MOESM1_ESM.zip › Supplementary File1/SSR_venn_diagram/GCF_009295925.1_ASM929592v1_genomic.fna_SSR.png]

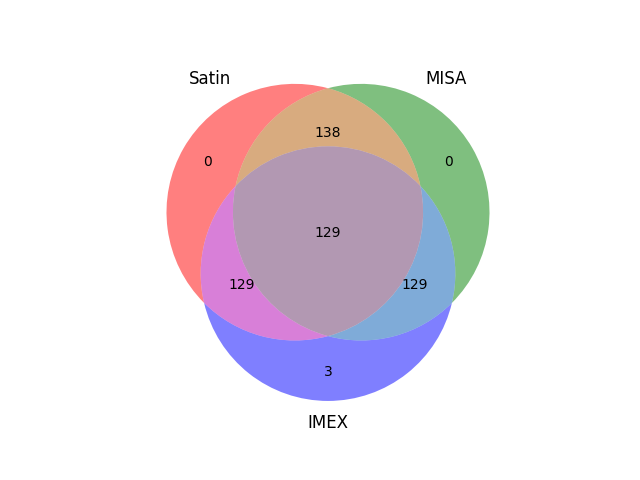

Supplement: Supplementary file 1 [file 12859_2024_5842_MOESM1_ESM.zip › Supplementary File1/SSR_venn_diagram/GCF_009729825.1_ASM972982v1_genomic.fna_SSR.png]

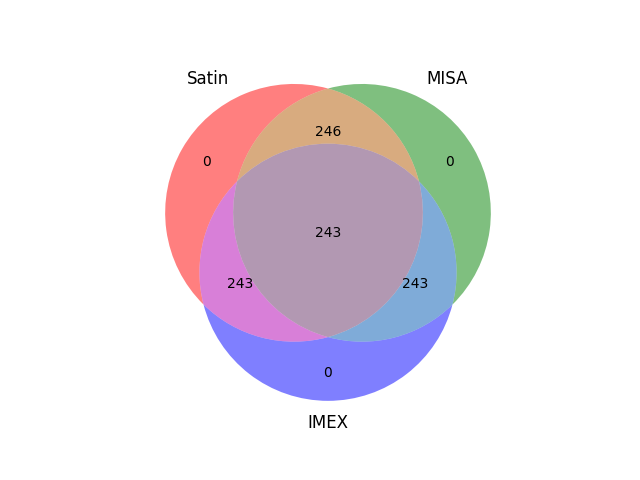

Supplement: Supplementary file 1 [file 12859_2024_5842_MOESM1_ESM.zip › Supplementary File1/SSR_venn_diagram/GCF_009789435.1_ASM978943v1_genomic.fna_SSR.png]

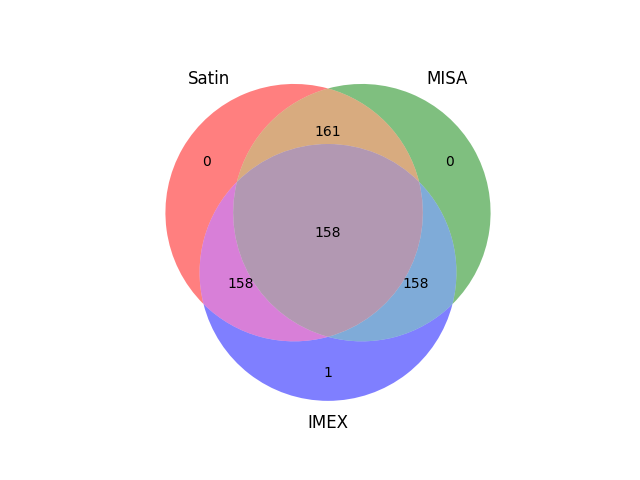

Supplement: Supplementary file 1 [file 12859_2024_5842_MOESM1_ESM.zip › Supplementary File1/SSR_venn_diagram/GCF_009798215.1_ASM979821v1_genomic.fna_SSR.png]

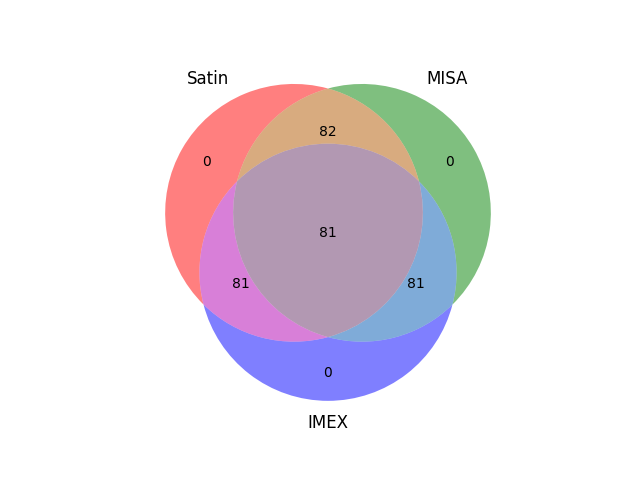

Supplement: Supplementary file 1 [file 12859_2024_5842_MOESM1_ESM.zip › Supplementary File1/SSR_venn_diagram/GCF_009814475.1_ASM981447v1_genomic.fna_SSR.png]

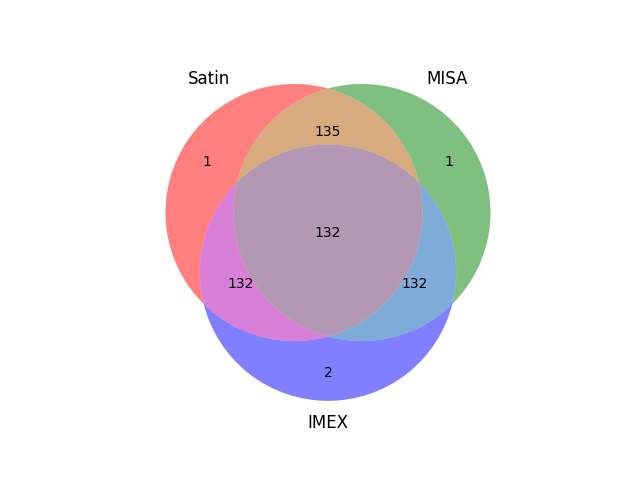

Supplement: Supplementary file 1 [file 12859_2024_5842_MOESM1_ESM.zip › Supplementary File1/SSR_venn_diagram/GCF_010571005.1_ASM1057100v1_genomic.fna_SSR.png]

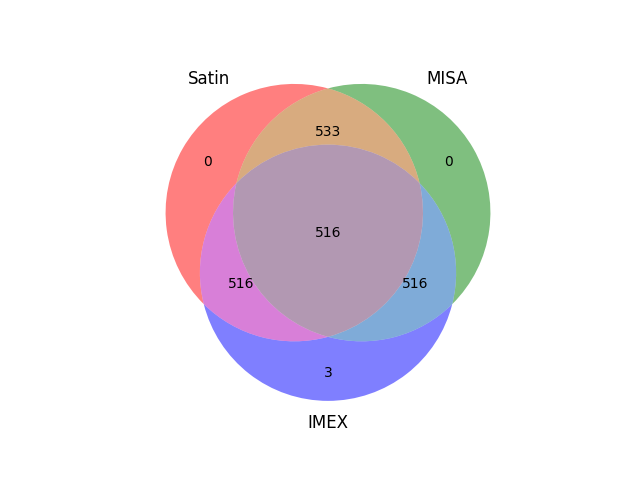

Supplement: Supplementary file 1 [file 12859_2024_5842_MOESM1_ESM.zip › Supplementary File1/SSR_venn_diagram/GCF_010729485.1_ASM1072948v1_genomic.fna_SSR.png]

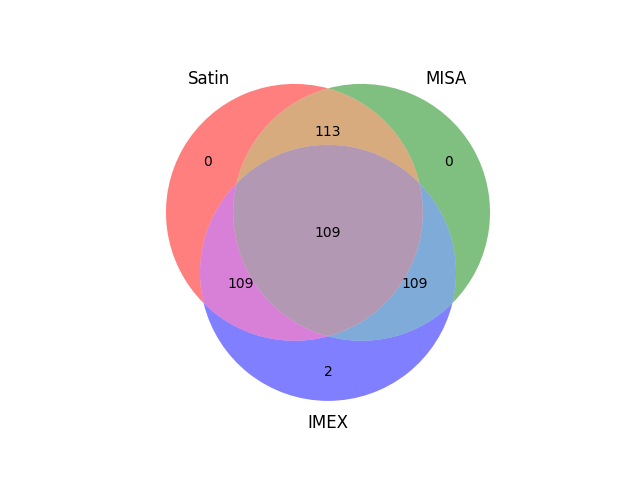

Supplement: Supplementary file 1 [file 12859_2024_5842_MOESM1_ESM.zip › Supplementary File1/SSR_venn_diagram/GCF_900051755.1_10625_3_79_genomic.fna_SSR.png]

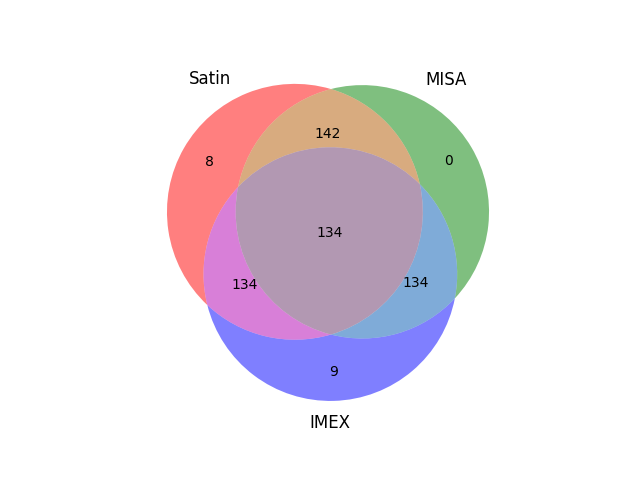

Supplement: Supplementary file 1 [file 12859_2024_5842_MOESM1_ESM.zip › Supplementary File1/SSR_venn_diagram/GCF_900082415.1_12673_4_43_genomic.fna_SSR.png]

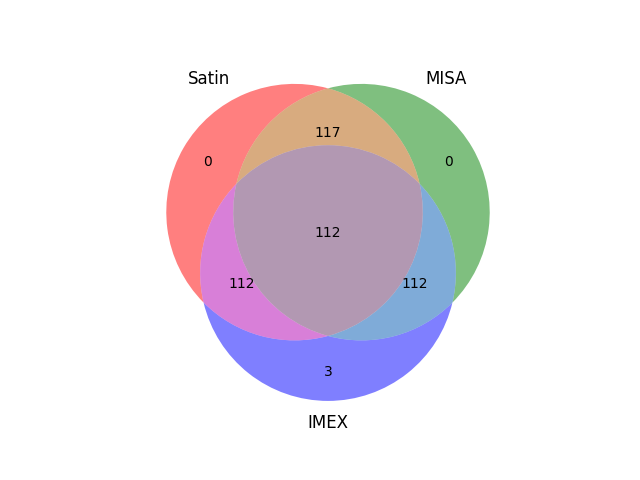

Supplement: Supplementary file 1 [file 12859_2024_5842_MOESM1_ESM.zip › Supplementary File1/SSR_venn_diagram/GCF_900088305.1_STT-1-2-15-Cm_genomic.fna_SSR.png]

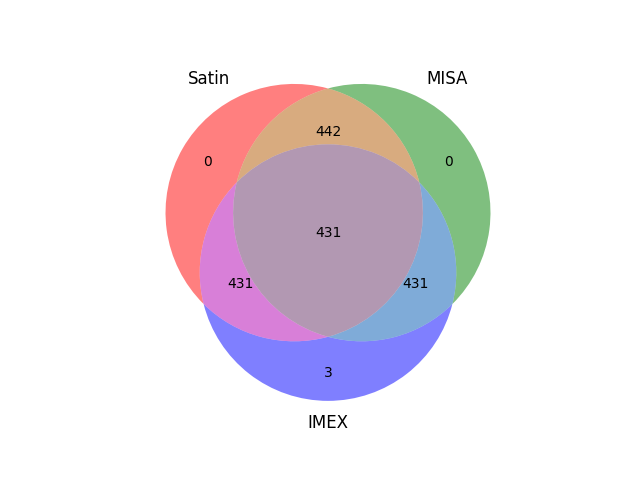

Supplement: Supplementary file 1 [file 12859_2024_5842_MOESM1_ESM.zip › Supplementary File1/SSR_venn_diagram/GCF_900098805.1_Microbacterium_sp._strain_Alg239_V18_genomic.fna_SSR.png]

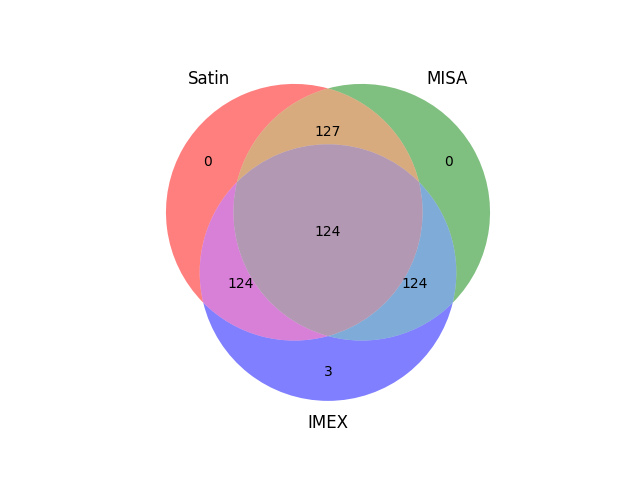

Supplement: Supplementary file 1 [file 12859_2024_5842_MOESM1_ESM.zip › Supplementary File1/SSR_venn_diagram/GCF_900102495.1_IMG-taxon_2671180042_annotated_assembly_genomic.fna_SSR.png]

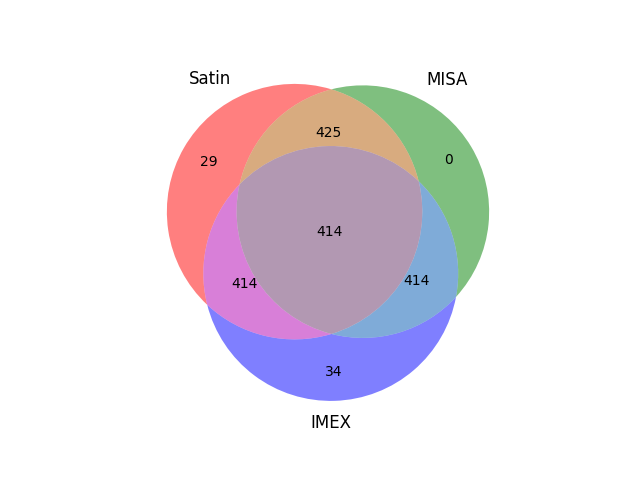

Supplement: Supplementary file 1 [file 12859_2024_5842_MOESM1_ESM.zip › Supplementary File1/SSR_venn_diagram/GCF_900122095.1_14722_7_86_genomic.fna_SSR.png]

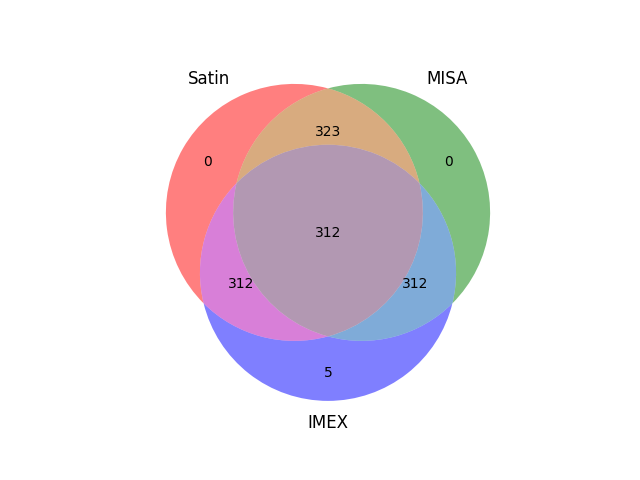

Supplement: Supplementary file 1 [file 12859_2024_5842_MOESM1_ESM.zip › Supplementary File1/SSR_venn_diagram/GCF_900131355.1_10665_3_82_genomic.fna_SSR.png]

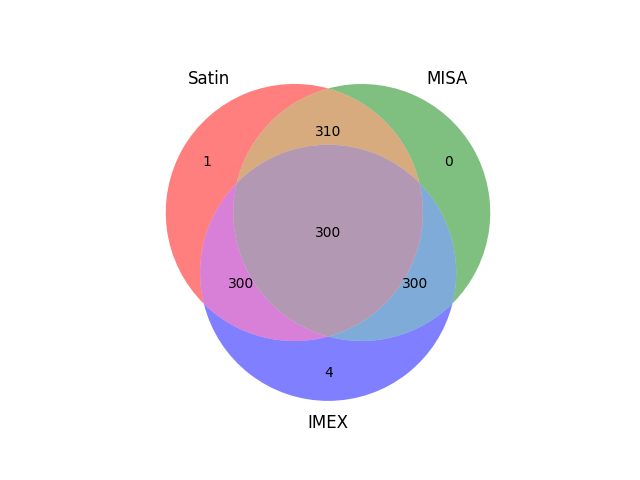

Supplement: Supplementary file 1 [file 12859_2024_5842_MOESM1_ESM.zip › Supplementary File1/SSR_venn_diagram/GCF_900136655.1_10625_5_22_genomic.fna_SSR.png]

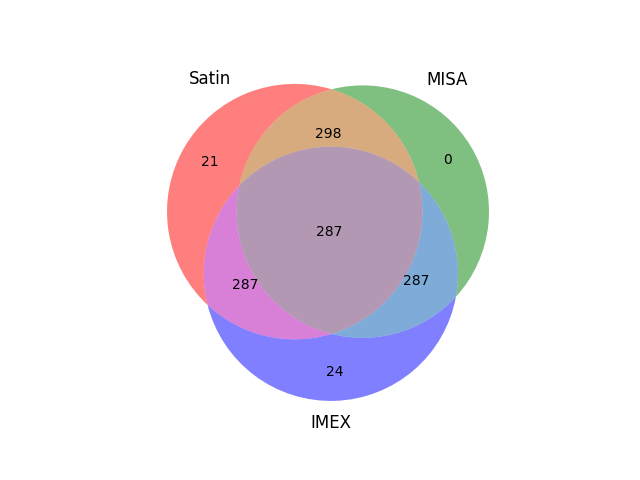

Supplement: Supplementary file 1 [file 12859_2024_5842_MOESM1_ESM.zip › Supplementary File1/SSR_venn_diagram/GCF_900138365.1_11893_7_50_genomic.fna_SSR.png]

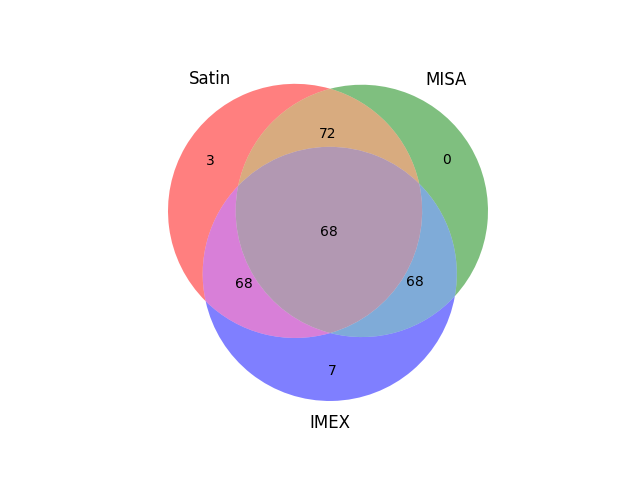

Supplement: Supplementary file 1 [file 12859_2024_5842_MOESM1_ESM.zip › Supplementary File1/SSR_venn_diagram/GCF_900171035.1_Hermans-1558_genomic.fna_SSR.png]

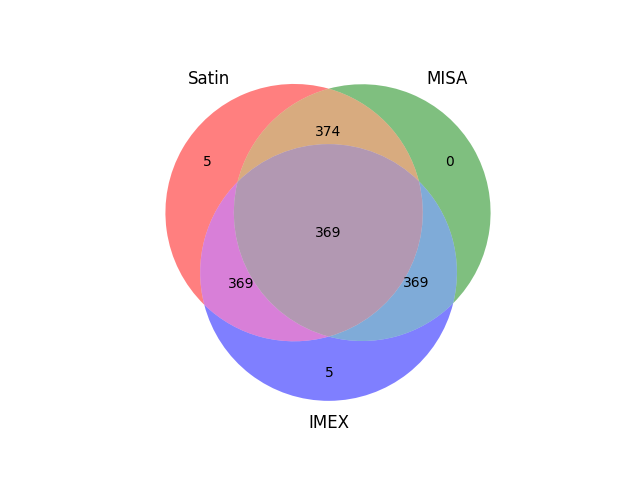

Supplement: Supplementary file 1 [file 12859_2024_5842_MOESM1_ESM.zip › Supplementary File1/SSR_venn_diagram/GCF_900173355.1_20794_7_175_genomic.fna_SSR.png]

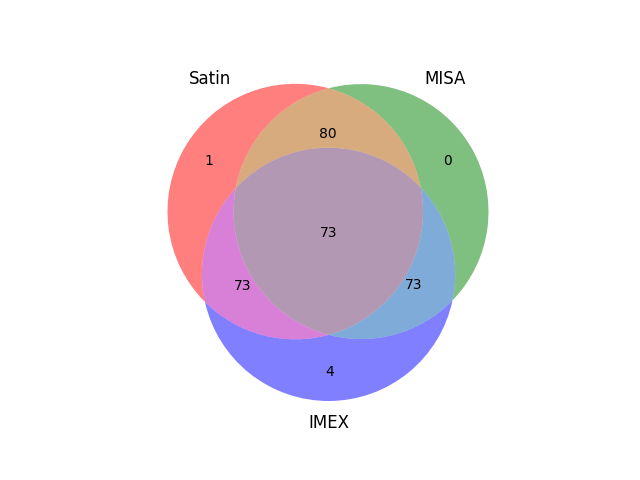

Supplement: Supplementary file 1 [file 12859_2024_5842_MOESM1_ESM.zip › Supplementary File1/SSR_venn_diagram/GCF_900189265.1_6470_5_13_genomic.fna_SSR.png]

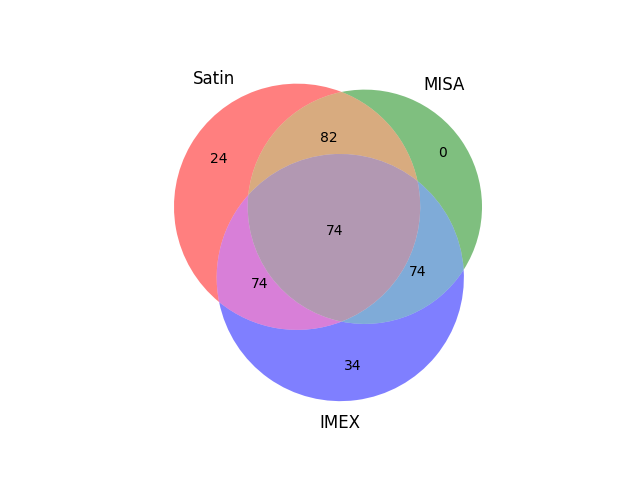

Supplement: Supplementary file 1 [file 12859_2024_5842_MOESM1_ESM.zip › Supplementary File1/SSR_venn_diagram/GCF_900194785.1_13681_2_4_genomic.fna_SSR.png]

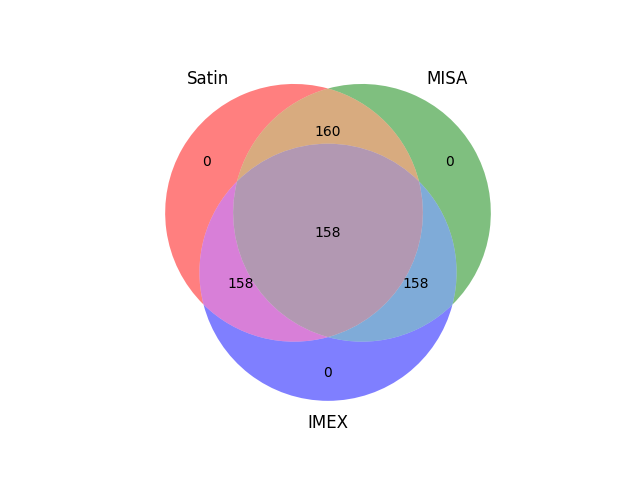

Supplement: Supplementary file 1 [file 12859_2024_5842_MOESM1_ESM.zip › Supplementary File1/SSR_venn_diagram/GCF_900196225.1_F2_55_genomic.fna_SSR.png]

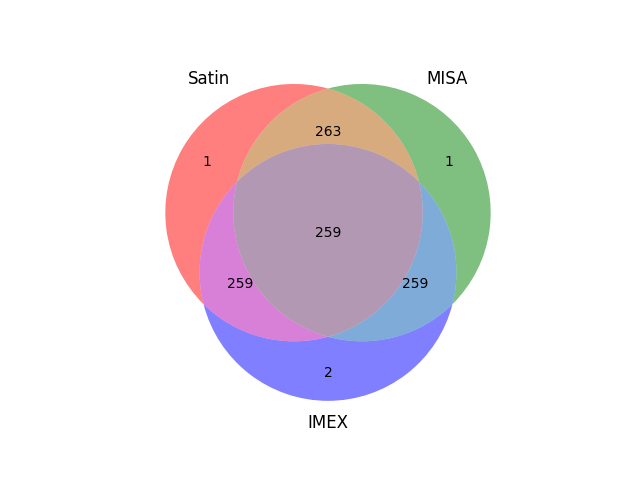

Supplement: Supplementary file 1 [file 12859_2024_5842_MOESM1_ESM.zip › Supplementary File1/SSR_venn_diagram/GCF_900242295.1_STY29_genomic.fna_SSR.png]

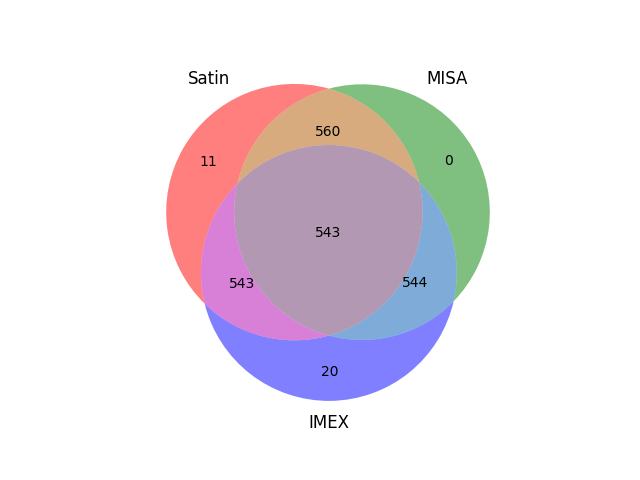

Supplement: Supplementary file 1 [file 12859_2024_5842_MOESM1_ESM.zip › Supplementary File1/SSR_venn_diagram/GCF_900380115.1_PD_4314_genomic.fna_SSR.png]

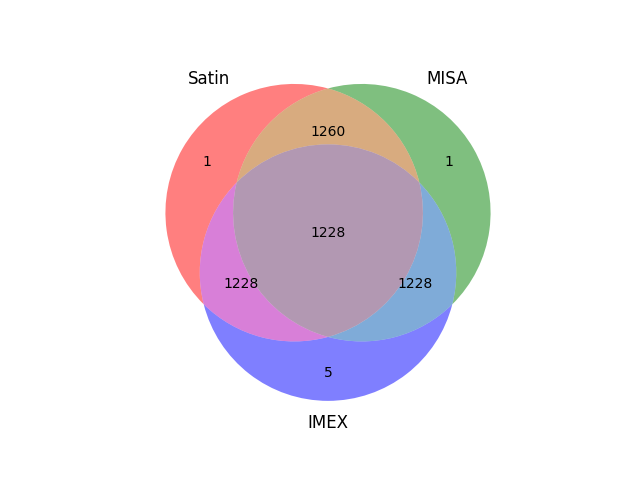

Supplement: Supplementary file 1 [file 12859_2024_5842_MOESM1_ESM.zip › Supplementary File1/SSR_venn_diagram/GCF_900445785.1_52451_D01_genomic.fna_SSR.png]

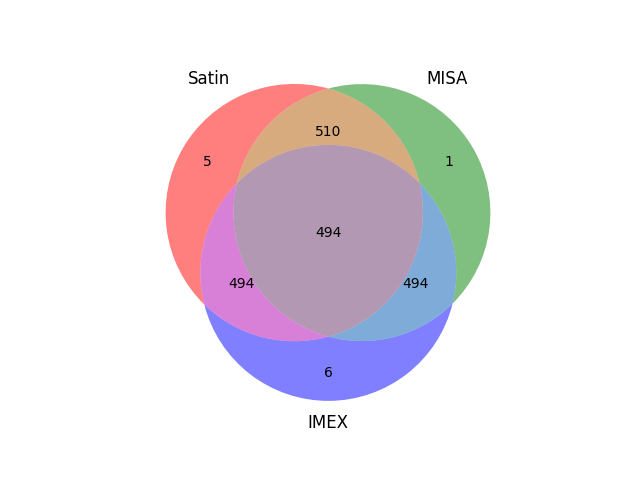

Supplement: Supplementary file 1 [file 12859_2024_5842_MOESM1_ESM.zip › Supplementary File1/SSR_venn_diagram/GCF_900471605.1_AFS068226_genomic.fna_SSR.png]

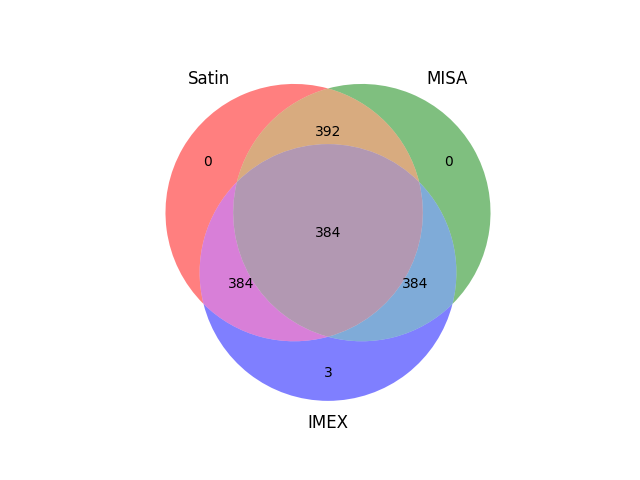

Supplement: Supplementary file 1 [file 12859_2024_5842_MOESM1_ESM.zip › Supplementary File1/SSR_venn_diagram/GCF_900508965.1_19646_3_63_genomic.fna_SSR.png]

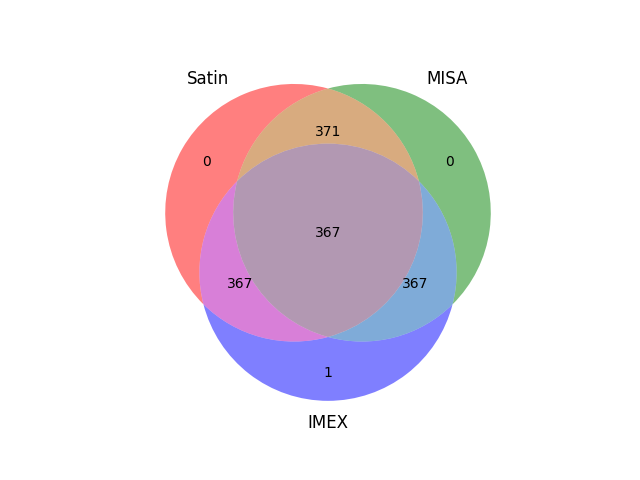

Supplement: Supplementary file 1 [file 12859_2024_5842_MOESM1_ESM.zip › Supplementary File1/SSR_venn_diagram/GCF_900513755.1_18174_5_54_genomic.fna_SSR.png]

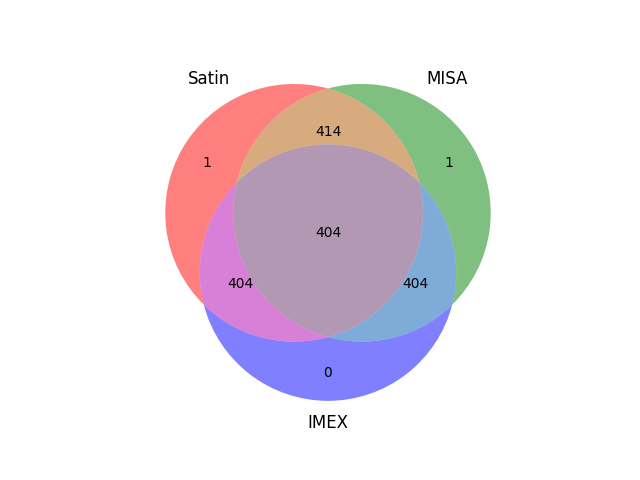

Supplement: Supplementary file 1 [file 12859_2024_5842_MOESM1_ESM.zip › Supplementary File1/SSR_venn_diagram/GCF_900688565.1_7614_7_47_genomic.fna_SSR.png]

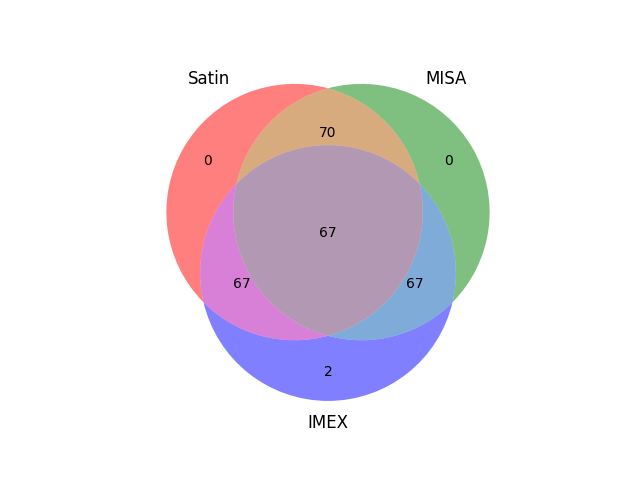

Supplement: Supplementary file 1 [file 12859_2024_5842_MOESM1_ESM.zip › Supplementary File1/SSR_venn_diagram/GCF_900989155.1_8849_2_45_genomic.fna_SSR.png]
